# Super‐Resolution Compatible DNA Labeling Technique Reveals Chromatin Mobility and Organization Changes During Differentiation

**DOI:** 10.1002/advs.202505955

**Published:** 2025-09-09

**Authors:** Maruthi K. Pabba, Miroslav Kuba, Tomáš Kraus, Kerem Celikay, Janis Meyer, Sunil Kumar Pradhan, Andreas Maiser, Hartmann Harz, Heinrich Leonhardt, Karl Rohr, Michal Hocek, M. Cristina Cardoso

**Affiliations:** ^1^ Cell Biology and Epigenetics Department of Biology Technical University of Darmstadt 64287 Darmstadt Germany; ^2^ Institute of Organic Chemistry and Biochemistry Czech Academy of Sciences Prague 6 CZ‐16000 Czech Republic; ^3^ Department of Organic Chemistry Faculty of Science Charles University Prague 2 CZ‐12843 Czech Republic; ^4^ Biomedical Computer Vision Group BioQuant IPMB Heidelberg University Baden‐Würtenberg 69120 Heidelberg Germany; ^5^ Human Biology and Bioimaging Faculty of Biology Ludwig Maximilians University Munich 81377 Munich Germany

**Keywords:** chromatin mobility, dCSiRTP, human iPSC differentiation, SNTT1, STED

## Abstract

Chromatin dynamics play a crucial role in cellular differentiation, yet tools for studying global chromatin mobility in living cells remain limited. Here, a novel probe is developeded for the metabolic labeling of chromatin and tracking its mobility during neural differentiation. The labeling system utilizes a newly developed silicon rhodamine‐conjugated deoxycytidine triphosphate (dC^SiR^TP). It is shown that this dCTP is efficiently delivered into living human induced pluripotent stem cells (iPSCs) and neural stem cells (NSCs) via a synthetic transporter (SNTT1). Using correlative confocal microscopy and stimulated emission depletion (STED) super‐resolution microscopy, the sizes of labeled chromatin domains are quantified. Time‐lapse super‐resolution microscopy combined with single particle tracking revealed that chromatin mobility decreases during the transition from iPSCs (pluripotent state) to NSCs and neurons (differentiated state). This reduction in mobility correlates with the differentiation state, reflecting changes in chromatin organization during cell fate commitment. Concomitant mechanistic insights obtained from micrococcal nuclease digestion assays, chromatin compaction, and histone modification analyses revealed a decrease in chromatin accessibility during neuronal differentiation. These data indicate that chromatin adopts a more constrained structure with reduced accessibility and increased heterochromatin‐associated histone modifications. These findings provide new insights into chromatin regulation during neurogenesis.

## Graphical abstract

A novel chromatin‐labeling probe is developed to investigate chromatin mobility during differentiation. The silicon rhodamine–conjugated dCTP is efficiently delivered into iPSCs and NSCs using a synthetic transporter. Super‐resolution microscopy and single‐particle tracking show decreased chromatin mobility during differentiation. Complementary assays confirm reduced accessibility and increased heterochromatin features, highlighting structural reorganization of chromatin during neurogenesis.

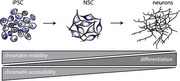

## 1 Introduction

The 3D organization and dynamics of chromatin play crucial roles in regulating gene expression and cellular identity.^[^ ^1^, ^2^ ^]^ While the structural aspects of chromatin organization have been extensively studied through techniques like Hi‐C and microscopy, our understanding of real‐time chromatin mobility during cellular differentiation remains limited.^[^ ^3^, ^4^ ^]^ This dynamic behavior of chromatin is particularly relevant in the context of cellular differentiation, where large‐scale reorganization of the genome occurs to establish and maintain cell type‐specific gene expression programs.^[^ ^5^ ^]^ Multiple studies have established the fundamental changes in chromatin structure during cellular differentiation.^[^ ^6^, ^7^, ^8^ ^]^ Particularly in neural development, epigenomic reprogramming involving chromatin accessibility, histone modifications, and 3D chromatin architecture has been extensively documented.^[^ ^9^ ^]^ These studies have established that developmental processes are accompanied by progressive chromatin compaction (characterized by reduced accessibility to nucleases and increased heterochromatin marks), though direct visualization of these dynamics in living cells has remained challenging.

The differentiation of human induced pluripotent stem cells (iPSCs) into neurons represents an excellent model system to study chromatin dynamics during cell fate transitions. Pluripotent stem cells are characterized by a unique nuclear architecture, featuring a more open and accessible chromatin landscape that is thought to contribute to their developmental flexibility, with the capacity to differentiate into a plethora of cell lineages.^[^ ^10^, ^11^ ^]^ During neural differentiation, dramatic changes in chromatin architecture have been documented. These include the formation of new topologically associating domains (TADs), the establishment of neural‐specific enhancer‐promoter interactions, and the separation of neuronal lineage specific genes from the nuclear lamina.^[^ ^12^, ^13^ ^]^

Recent single‐nucleosome imaging has revealed that chromatin exhibits continuous motion with distinct organizational states in living cells.^[^ ^14^, ^15^, ^16^ ^]^ Chromatin mobility serves as a sensitive marker of cellular differentiation, with studies showing transitions from high‐mobility pluripotent states to more constrained differentiated states.^[^ ^17^, ^18^ ^]^ These mobility differences reflect distinct chromatin states and regulatory factor dynamics.^[^ ^19^, ^20^ ^]^

The local diffusion of chromatin structures within the nucleus has emerged as a parameter that may influence gene regulatory processes.^[^ ^21^, ^22^, ^23^, ^24^, ^25^, ^26^ ^]^ Recent studies using various particle‐tracking approaches have revealed that chromatin displays complex motion patterns that can be classified into multiple distinct models, including confined diffusion, directed motion, and anomalous diffusion.^[^ ^22^, ^23^, ^27^ ^]^ Importantly, the relationship between chromatin mobility and compaction appears more complex than initially assumed, with some studies suggesting that mobility can be uncoupled from compaction in certain cellular contexts.^[^ ^23^ ^]^

Single‐cell studies have revealed unprecedented heterogeneity in chromatin states during neural differentiation, with distinct chromatin accessibility patterns emerging even before fate‐determining transcriptional programs are activated.^[^ ^28^ ^]^ Advanced imaging techniques have shown that chromatin compaction states correlate strongly with cell fate commitment, where regions containing neural‐specific genes undergo significant structural reorganization during differentiation.^[^ ^29^ ^]^ Notably, disruption of chromatin remodeling factors during neural differentiation can lead to severe neurodevelopmental disorders, highlighting the critical importance of proper chromatin dynamics in neural development.^[^ ^30^ ^]^ Recent developments in live‐cell imaging have enabled visualization of chromatin dynamics with unprecedented temporal resolution, revealing rapid local reorganization events that occur on timescales of seconds to minutes.^[^ ^31^, ^32^, ^33^ ^]^ Previous studies have established that histone exchange changes during cellular differentiation across multiple cell types. It has been shown that architectural chromatin proteins exhibit hyperdynamic binding in pluripotent embryonic stem cells and become immobilized upon differentiation.^[^ ^10^ ^]^ Chromatin dynamics decrease during muscle differentiation, with nuclear protein mobility serving as a differentiation index.^[^ ^18^ ^]^ More recently, studies have demonstrated that chromatin undergoes a sol‐gel transition during differentiation.^[^ ^17^ ^]^ However, most of these studies quantified individual histone protein dynamics using fluorescence photobleaching analysis, while the mobility behavior of chromatin domains themselves during human neural differentiation remains unclear.

Studies have used artificial DNA sequences in genomic loci and used a large array of chromatin binding proteins to visualize the loci, chromatin dynamics may be altered.^[^ ^34^ ^]^ Therefore, a more direct way to measure chromatin dynamics is to label and track the DNA directly.^[^ ^35^ ^]^ Stable fluorescent labeling of DNA is essential for tracking dynamic changes in chromatin components *in cellulo*. Intercalative dyes, while readily available, are unsuitable due to their non‐specific, homogeneous staining of all DNA and their toxic effects. Metabolic labeling of DNA, in contrast, offers both stability, through covalent attachment of fluorophores, and topological specificity, by labeling only replicating DNA segments. This allows for the subsequent precise segmentation and image analysis. Various fluorescent tags and incorporation strategies have been developed for covalent DNA labeling.^[^ ^36^ ^]^ In vitro enzymatic synthesis of fluorescently labeled DNA using modified deoxyribonucleoside triphosphates (dNTPs) leverages the tolerance of non‐natural DNA polymerases (e.g., KlenTaq, KOD, and Vent(exo‐)) toward these modified substrates.^[^ ^37^, ^38^ ^]^ However, *in cellulo*, metabolic labeling is significantly more challenging.^[^ ^39^, ^40^ ^]^ The anionic nature of dNTPs prevents their penetration across cell membranes, with rare exceptions.^[^ ^41^ ^]^ Conversely, cell‐permeable modified nucleosides are often poor substrates for endogenous kinases, hindering their phosphorylation and subsequent incorporation into DNA.^[^ ^39^ ^]^ This “deadlock,” which undoubtedly serves as a natural safeguard for the genetic code, has been circumvented by various “chemical” strategies, each with its own advantages and limitations in live‐cell applications.

Traditional methods^[^ ^42^ ^]^ utilizing 5‐halogenated uridines (e.g., CldU, BrdU, IdU) exploit their membrane permeability and compatibility with cellular enzymatic machinery. However, the requirement for cell fixation and secondary labeling with fluorescent antibodies precludes their use in live‐cell imaging.^[^ ^39^, ^40^ ^]^ Click‐chemistry approaches, designed to eliminate laborious immunostaining, employ nucleosides with small reactive “handles” like ethynyl or vinyl groups; these nucleosides are endogenously phosphorylated and incorporated into genomic DNA. Cu(I)‐catalyzed azide‐alkyne cycloaddition (CuAAC) using 5‐ethynyl‐2′‐deoxyuridine (EdU) and azide‐functionalized fluorophores^[^ ^43^ ^]^ has become a widely adopted technique in cell biology, but still necessitates cell fixation, precluding live‐cell imaging. Copper‐free alternatives, such as inverse electron‐demand Diels‐Alder (IEDDA) reactions, have been explored using 5‐vinyl‐2′‐deoxyuridine (VdU) as a DNA reporter in replicating cells,^[^ ^44^, ^45^, ^46^, ^47^ ^]^ including light‐induced variants.^[^ ^48^, ^49^, ^50^ ^]^ Strain‐promoted azide‐alkyne cycloadditions (SPAAC) with azide‐substituted deoxyadenosine have also been utilized.^[^ ^51^ ^]^ Despite significant advancements in IEDDA and SPAAC labeling, these two‐step procedures still face practical limitations for routine cell biology applications. These include prolonged incubation times with the primary nucleoside (15–48 h),^[^ ^45^, ^50^ ^]^ cytotoxicity,^[^ ^46^ ^]^ and low incorporation efficiency under standard culture conditions.^[^ ^47^ ^]^

Since phosphorylation often represents the bottleneck in the DNA labeling pathway, direct delivery of fluorescently labeled dNTPs has been attempted. Mechanical^[^ ^35^, ^52^, ^53^ ^]^ and electroporation^[^ ^54^ ^]^ methods have enabled rapid delivery of modified dNTPs and, in some cases, incorporation into genomic DNA. However, these techniques lack versatility for high‐throughput experiments involving diverse cell types, both adherent and suspension. The TriPPro approach, employing protective groups on the triphosphate moiety of 2‐TCO‐modified 2′‐deoxycytidine triphosphate, enabled visualization of *de novo* synthesized cellular and viral DNA via IEDDA with cell‐permeable fluorescent dye‐tetrazine conjugates.^[^ ^55^ ^]^ Rapid intracellular DNA labeling has also been achieved with TAMRA‐dATP, though this appears to be an exceptional case of unassisted delivery.^[^ ^41^ ^]^

The development of synthetic nucleoside triphosphate transporters (SNTTs) capable of delivering modified dNTPs has provided novel tools for labelling chromatin while maintaining cellular viability. SNTT1 facilitates the rapid delivery of various labeled dNTPs into various types of live‐cells. The incorporation of fluorescently labeled dNTPs into genomic DNA proceeds within minutes, and fluorescent labeling is achieved either via a single‐step^[^ ^56^, ^57^, ^58^, ^59^, ^60^ ^]^ or a two‐step procedure.^[^ ^61^ ^]^ However, our previous efforts to develop a labeled dNTP with a far‐red fluorophore suitable for STED microscopy have been only partially successful due to either low incorporation efficiency^[^ ^59^ ^]^ or cytotoxicity.^[^ ^62^ ^]^

Here, we present a novel approach to study global chromatin structure and dynamics throughout neural differentiation in human cells. Our newly developed dCSiRTP nucleotide, delivered via SNTT1, overcomes the limitations of our prior work by achieving high DNA‐incorporation efficiency in metabolic labeling of stem cells with a far‐red emitting fluorophore. This technological advance enabled us to employ live‐cell super‐resolution STED microscopy, tracking the dynamics of chromatin structures with unprecedented resolution throughout the entire process of neural differentiation.

## 2 Results and Discussion

### 2.1 Synthesis of STED‐Compatible Fluorescently Labeled Nucleotides and Labeling of Chromatin

**Figure 1:**
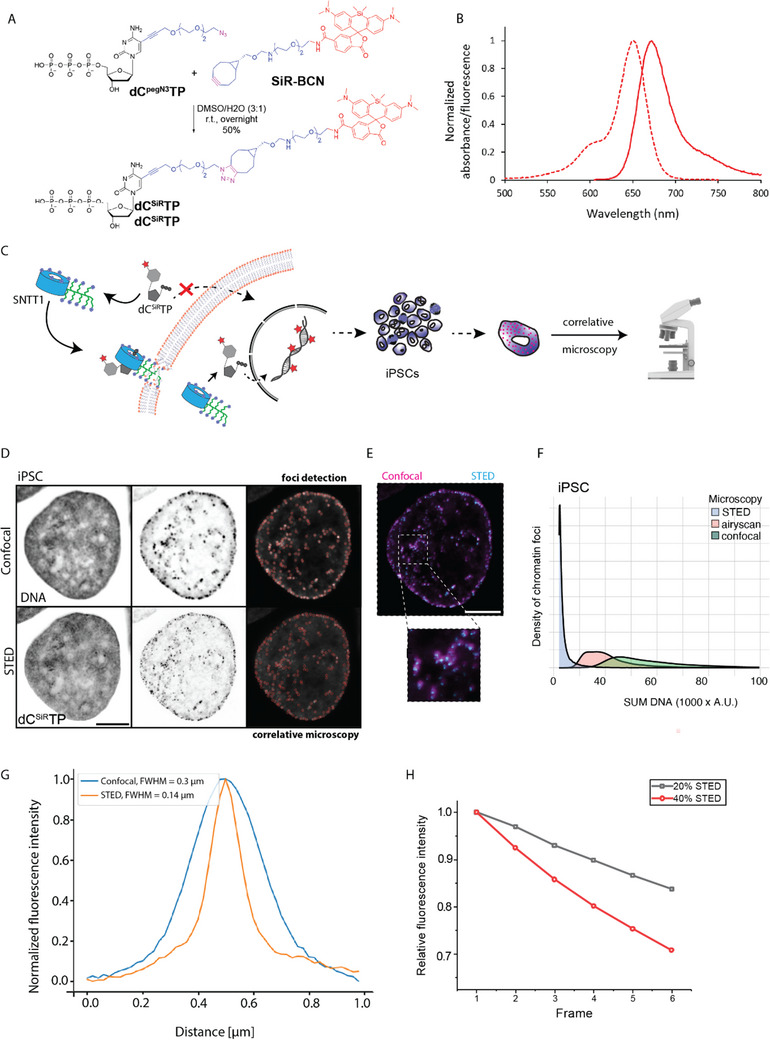
Synthesis and chromatin labeling using dC^SiR^TP, A) Chemical synthesis and metabolic DNA labeling in living cells. SiR‐BCN click reaction with azido‐terminated dC^pegN3^TP precursor. B) Absorption and fluorescence spectrum of DNA19_1C^SiR^ in PBS. C) A schematic representation of transport of dC^SiR^TP across the cell membrane by synthetic nucleoside triphosphate transporter (SNTT1) and subsequent visualization of labeled DNA foci by correlative microscopy. D–F) Chromatin labeled with dC^SiR^TP and imaged using correlative microscopy including stimulated emission depletion (STED) microscopy, confocal microscopy and Airyscan joint deconvolution microscopy (Table S6, Supporting Information). The representative images show the total DNA and dC^SiR^TP labeled chromatin sites in grey scale and overlay image of dC^SiR^TP foci in cyan – STED, magenta – confocal. The SUM DNA (total DNA content) was plotted for different spatial resolutions. Scale bars: 5 µm. (G) Normalized averaged fluorescence intensity profiles for foci in confocal (blue) and STED (orange) microscopy. Full Width at Half Maximum (FWHM) values are provided in the figure legend. (H) The bleaching property over time was plotted as relative fluorescence intensity for SiR‐dCTP with different laser powers.

Silicon rhodamine‐modified nucleoside triphosphate dC^SiR^TP was prepared by strain‐promoted azide‐alkyne cycloaddition reaction of azido‐propargyloxy‐triethylene glycol‐conjugated deoxycytidine triphosphate dC^pegN3^TP^[^ ^63^ ^]^ with bicyclononyne‐linked silicon rhodamine fluorophore SiR‐BCN (**Figure** **1**A). The product was isolated in 50% yield after purification by reverse‐phase high‐performance liquid chromatography (HPLC). The solution of modified nucleotide dC^SiR^TP in phosphate buffered saline (PBS) showed an absorption maximum and an emission maximum at 651 and 672 nm, respectively (Table S1 and Figure S1A, Supporting Information). The corresponding extinction coefficient and quantum yield in PBS were calculated to be 124 800 M^−1^cm^−1^ and 54%, respectively.

The modified dC^SiR^TP was tested as a substrate for KOD XL DNA polymerase in primer extension (PEX) and polymerase chain reaction (PCR) experiments. PEX with 19‐mer (Table S2, Supporting Information) encoding for incorporation of a single modification proceeded smoothly and the product was detected by polyacrylamide gel electrophoresis (PAGE) (Figure S2A, Supporting Information) and by MALDI‐TOF mass spectrometry analysis (Figures S3–S7, Supporting Information). The SiR‐labeled DNA in PBS showed an absorption maximum and an emission maximum at 651 and 672 nm, respectively (Figure 1B), with a quantum yield of 48% (Table S1, Supporting Information). Thus, no significant change in the photophysical properties of the SiR fluorophore was observed. PCR was tested using mixtures with varying ratios of dC^SiR^TP and dCTP (0‐100%). Amplicon formation was detectable with up to 60% of dC^SiR^TP used in the reaction mixture. (Figure S2B and see Tables S2, S3, Supporting Information for primer and template sequences).

Using the modified dC^SiR^TP nucleotide, with a STED‐compatible (super‐resolution microscopy) fluorophore, we proceeded to test it on cultures of human induced pluripotent stem cells (iPSCs) (Table S4, Supporting Information). First, the iPSCs were checked for pluripotency using immunofluorescence detection of the pluripotent markers Sox2 and Oct 3/4, and active DNA replication was verified using incorporation and detection of EdU. The iPSC cultures were positive for pluripotent markers and were actively replicating the genomic DNA (Figure S8, Supporting Information).

Subsequently, we labeled chromatin by incubating cells in a tricine buffer (pH 7.4) containing 10 µM SNTT1 and 10 µM dC^SiR^TP for 15 min at 37 °C (Table S5, Supporting Information). SNTT1 facilitated the transport of labeled nucleotides into the cells, allowing for an efficient incorporation of the labeled nucleotides into the genome of the replicating cells. This method has proven effective for the rapid labeling of DNA in cells without altering their native chromatin state. By directly labeling chromatin/DNA in replicating S phase cells, we can label any chromatin type (euchromatin, facultative heterochromatin, constitutive heterochromatin) as well as DNA repeat elements like LINEs and SINEs, as well as tandem repeats, which are overlooked in most studies. Using directly labeled deoxyribonucleotides we can label the DNA genome‐wide and examine chromatin dynamics in its native state over several cell cycles. As a larger portion of genomic DNA is packaged into heterochromatin, this would also be reflected by this labeling method. Using the SNTT1 has allowed us to perform rapid chromatin labeling (within 10–15 min) without disrupting cell morphology by avoiding the trypsinization required by electroporation‐based intracellular delivery methods. This allowed immediate imaging (Movie S1, Supporting Information). After labeling chromatin in live‐cells with dC^SiR^TP and staining of total DNA with a live‐cell DNA dye, the iPSCs were fixed for correlative microscopic imaging. The fluorescently labeled chromatin structures we track represent DNA segments labeled with dC^SiR^TP during DNA replication, which we term “chromatin domains” hereafter. These domains represent stable 3D folded structures of newly replicated DNA.

Cells with labeled chromatin (dC^SiR^TP) were imaged, comparing different spatial resolution techniques including STED microscopy, laser scanning confocal microscopy, and Airy scan microscopy (Figure 1C; Table S6, Supporting Information). Representative gray‐scale images of the DNA dye (Abberior 590 DNA dye) and dC^SiR^TP obtained from both confocal and STED imaging are shown in Figure 1D. The overlay image (Figure 1E) displays the same labeled chromatin visualized with STED (cyan) and confocal (magenta) microscopy. We observed that each clustered focus detected by confocal microscopy contained multiple, distinct foci when resolved with STED microscopy (Figure 1E). We then segmented the labeled foci at different spatial resolutions and plotted (Figure 1F) the foci count (y‐axis: density) against the total DNA content within the segmented foci (x‐axis: SUM DNA). The results demonstrated that STED microscopy provided significantly higher resolution of chromatin structure compared to confocal microscopy and Airy scan imaging. Additionally, to quantify the resolution gain between STED and confocal microscopy, a Full Width at Half Maximum (FWHM) analysis of the normalized fluorescence intensity profiles was performed (Figure 1G). We observed that the average measured values of FWHM for STED and confocal microscopy are 0.14 and 0.3 µm, respectively. Thus, there is a significant resolution gain in STED microscopy (Methods: resolution gain analysis of STED microscopy). Moreover, we quantified the bleaching properties of dC^SiR^TP using 20% and 40% full STED laser power levels over time, which showed that the fluorescent nucleotides are quite resistant to photobleaching (Figure 1H).

### 2.2 Chromatin Mobility Decreases Upon Neural Differentiation

**Figure 2:**
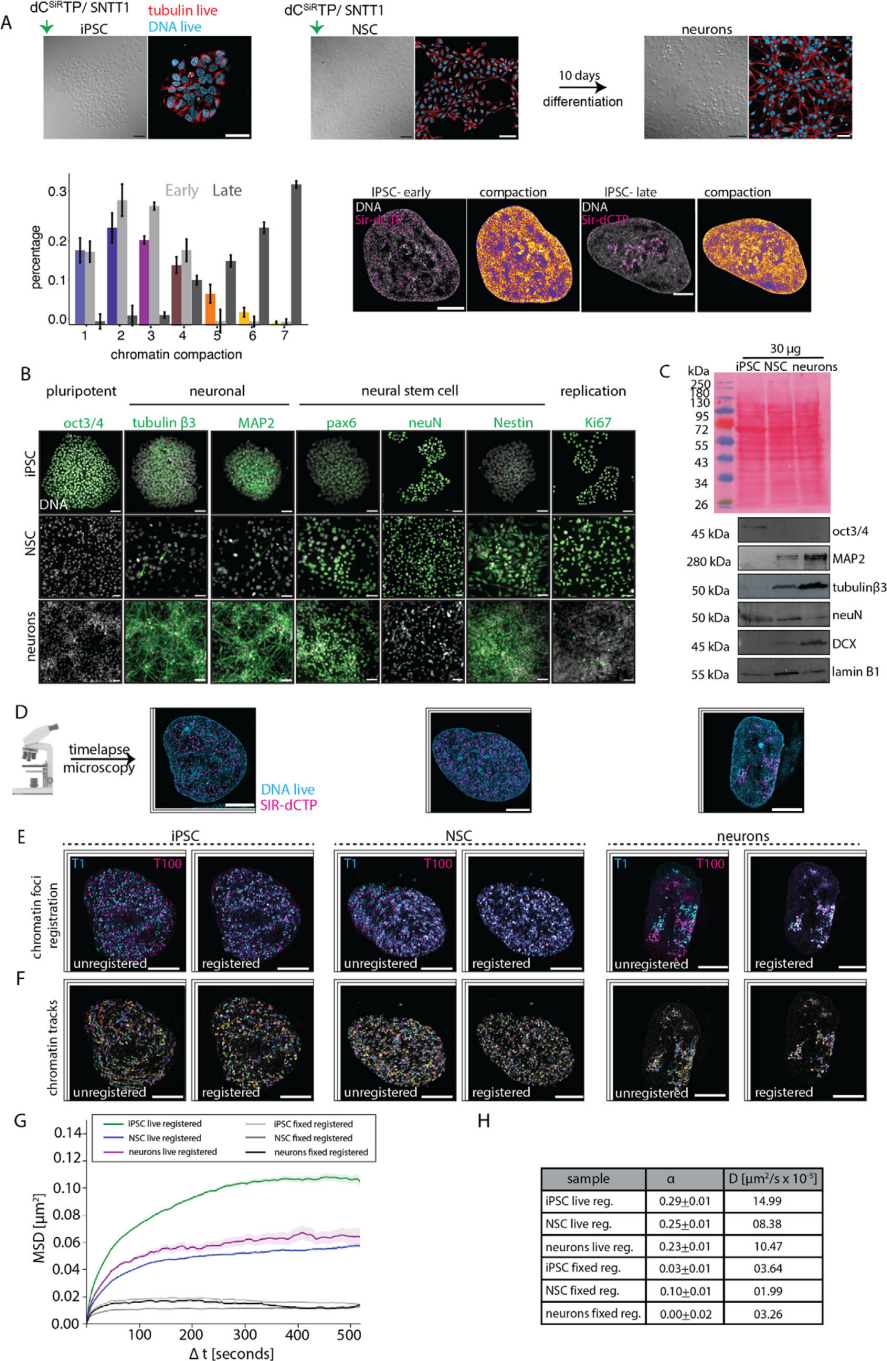
Characterization and chromatin mobility changes during neuronal differentiation. A) Differential interference contrast (DIC) and tubulin (red) + DNA (cyan) live‐cell dye images for iPSC, NSC and neurons. Scale bars: 50 µm (black) and 10 µm (white). Chromatin compaction analysis of early and late replication patterns (dC^SiR^TP) of iPSC cells was plotted as a histogram. Class 1 corresponds to the less compacted chromatin and class 7 the highest compacted chromatin. The representative images show the chromatin labeled cells with DAPI (gray) and dC^SiR^TP(magenta) and heat map of chromatin compaction. B) Immunodetection of pluripotent and neuronal lineage specific markers in iPSC, NSC and neurons (Methods, Table S8, Supporting Information). Scale bars: 50 µm. C) Western blot detection of pluripotent and neuronal lineage specific markers in iPSC, NSC and neurons (2 biological replicates). D) Chromatin labeled iPSC, NSC and neurons using SNTT1 + dC^SiR^TP complex (DNA – cyan, dC^SiR^TP – magenta) (> 4 biological replicates). E) Labeled chromatin in all cell types was imaged using live‐cell microscopy and foci from time frame 1 (T1‐ cyan) was overlaid with time frame 100 (T100‐ magenta). Scale bars: 10 µm. F) Registration of chromatin was performed using the live‐cell DNA dye as reference to estimate the actual chromatin mobility. The tracks in unregistered versus registered movies over time are illustrated to show the importance of 3D live‐cell registration. G) Mean square displacement (y‐axis, µm^2^) over time (x‐axis, Δt in seconds) for iPSC (green), NSC (blue) and neurons (magenta). The fixed cells MSD (gray shade) for each cell differentiation state was plotted in grey. H) The diffusion constant (D – µm^2^/s x 10^−5^) and the ɑ values with corresponding standard deviations are listed.

We next performed in vitro differentiation of iPSCs to obtain precursor neural stem cells (NSCs) as described (method S6, Tables S4, S7 and Figure S9, Supporting Information). Then, we established a protocol for differentiation of NSCs to neurons (methods S6, Table S7, Supporting Information). Differential interference contrast (DIC) images of live‐cells, where NSCs were differentiated into neurons for 10 days, are shown in Figure S10 (Supporting Information). The DIC images clearly show the neuronal network showing successful neuronal differentiation (Figure S10, Supporting Information). To further visualize the cellular morphology of iPSCs, NSCs, and neurons, we acquired DIC images concomitantly with images of cells stained with the live‐cell DNA dye (cyan) and a tubulin live‐cell dye (red) (**Figure** **2**A). Clear morphological differences between the differentiated stages were evident with the double live‐cell dye staining.

To ensure no bias toward any specific type of chromatin (euchromatin or heterochromatin), we sorted cells into early and late replication patterns based on the dC^SiR^TP label replication pattern. We then classified the total DNA (DAPI) into seven different compaction classes based on intensities (Methods: chromatin compaction analysis). We further mapped the spatial location of the replication label within the chromatin compaction classes for early and late replication IPSC cells (Figure 2A). We observed that early replicating cells have the dC^SiR^TP intensities in classes 1–4, whereas late replicating cells have dC^SiR^TP intensities in classes 4–7. Therefore, by sorting the cells based on replication patterns, we ensured that there is no bias toward the type of chromatin within our next analysis steps.

To validate the in vitro differentiation of iPSCs into neurons, we performed immunofluorescence staining on iPSCs, NSCs, and neurons using specific markers for pluripotency, neural stem cells, and mature neurons (Figure 2B; Figures S11 and S12, Supporting Information). Immunostaining revealed that only iPSCs were positive for the pluripotency marker Oct3/4, whereas NSCs and neurons were not, confirming the loss of pluripotency factors during differentiation. Moreover, we observed that NSCs exclusively expressed neural stem cell markers such as NeuN, Nestin, and Pax6 (Figure 2B; Figures S11 and S12, Supporting Information). In contrast, neurons were positive for mature neuronal markers tubulin beta 3 and MAP2, while only a few NSCs were positive for these markers (Figure 2B; Figures S11 and S12, Supporting Information). We further corroborated these findings using western blot analysis of whole cell lysates from iPSCs, NSCs, and neurons. The western blot analysis included detection of Oct3/4, MAP2, tubulin beta III, NeuN, and DCX, with lamin B1 and Ponceau Red staining serving as a loading control (Table S8, Supporting Information; Figure 2C). The western blot results were consistent with the immunostaining findings, confirming the successful differentiation of iPSCs into neurons.

Having established the neuronal differentiation system, we aimed to determine whether chromatin dynamics are affected during differentiation. To investigate this, we cultured iPSCs and NSCs on coated high‐precision coverslips and labeled their chromatin using SNTT1 and dC^SiR^TP (Figure 2A). Following labeling, we replaced the buffer with growth medium and allowed the cells to recover for 1–2 h. This allowed us to label DNA/chromatin directly using fluorescently labelled nucleotides. These labeled chromatin domains/foci represent the fluorescently labeled DNA/chromatin structures folded in the 3D space.

After this period, both iPSCs and NSCs were imaged using Airyscan microscopy. For a subset of chromatin‐labeled NSCs, we replaced the medium with differentiation medium and cultured the cells for 10 days to induce differentiation into neurons before imaging. We then identified cells with chromatin labeled by dC^SiR^TP (magenta) and performed live‐cell time‐lapse microscopy with Airyscan microscopy using two channels: dC^SiR^TP (magenta) and a live DNA stain (cyan). Images were acquired using an Airyscan microscope (Zeiss) with 2 channels imaging and 3D z‐stacks (5 stacks per channel, x‐y: 48 nm and z–170 nm) with a frame interval of Δt = 5 s (Figure 2D; Movie S2, Supporting Information). Movie S2 (Supporting Information) illustrates cell movement and labeled chromatin mobility over time, along with nuclear shape changes over time. To compensate for cell movement and accurately measure chromatin mobility within the cell, we performed 3D image registration of the live‐cell time‐lapse movies. Representative images of labeled chromatin at the time point 1 (T1, cyan) and 100 (T100, magenta) of the time‐lapse were overlaid before and after registration (Δt = 5 s) (Figure 2E). In the unregistered image, we observed directed chromatin motion due to cell movement, an effect eliminated by the 3D image registration (Figure 2E). This effect is further visualized by comparing chromatin tracks before and after registration (Figure 2F). We observed that after image registration, the chromatin tracks (random colors) lost their “apparent” directed motion over time, as clearly seen in Movie S3 (Supporting Information) and Figure 2F. Subsequently, we performed detection and tracking of the 3D chromatin foci followed by mean square displacement (MSD) analysis on the registered time‐lapses for iPSCs (green), NSCs (blue), and neurons (magenta), and plotted the results (Figure 2G). Generally, a higher MSD curve indicates greater chromatin diffusivity. We investigated whether different foci sizes corresponded to differences in diffusivity (Figure S13A,B,C, Supporting Information). For iPSC, the large foci exhibit somewhat less motion than the small foci. For NSC, we observed basically no differences. For the neurons, some differences could be detected, which may be attributed to a much lower number of trajectories with more noise. Interestingly, the volume of foci increases significantly during differentiation, suggesting that the labeled chromatin domains get larger (Figure S13A,B, Supporting Information).

Previous studies have shown that chromatin undergoes constrained diffusional motion,^[^ ^64^ ^]^ yielding a characteristic MSD curve, which we observe in our data. Therefore, we used a standard diffusion model that is fitted to the linear part at the beginning of the MSD curves to determine diffusion rates (short‐range diffusion). We observed (Figure 2G,H) that chromatin in iPSCs (14.99 × 10^−5^ µm^2^ s^−1^) is more mobile than in NSCs (8.38 × 10^−5^ µm^2^ s^−1^) and neurons (10.47 × 10^−5^ µm^2^ s^−1^). As a control for imaging system vibrations, we performed time‐lapse microscopy on formaldehyde‐fixed labeled cells. As shown in Movie S4 (Supporting Information), these fixed cells exhibit minimal motion or diffusion. Interestingly, NSCs and neurons showed similar diffusion rates, with chromatin in neurons appearing slightly more mobile than in NSCs (Figure 2G,H). Previous studies have used RNA seq and ATAC seq to understand the changes in chromatin during neuronal differentiation^[^ ^65^ ^]^ and have shown chromatin accessibility and transcription profiles are altered during differentiation. It is interesting to speculate whether this reduction in chromatin mobility correlates with compositional changes in chromatin remodeling complexes from pluripotency‐associated esBAF to neural‐specific nBAF complexes,^[^ ^66^ ^]^ expansion of lamin‐associated domains that physically anchor chromatin to the nuclear envelope,^[^ ^67^ ^]^ and HP1α‐mediated heterochromatin formation through phase separation.^[^ ^68^ ^]^ Our analysis reveals that the pluripotent state has higher chromatin mobility, and this decreases upon neural differentiation to NSCs and neurons, raising the question of whether global chromatin structural change, including altered histone modifications and reduced nuclease accessibility, contributes to these observed dynamics. Our findings are consistent with previous studies demonstrating chromatin mobility changes during differentiation^[^ ^10^, ^17^, ^18^ ^]^ but extend this concept by providing direct DNA labeling measurements during human neural differentiation.

### 2.3 Chromatin State Changes During Differentiation Correlate with Its Dynamics

**Figure 3:**
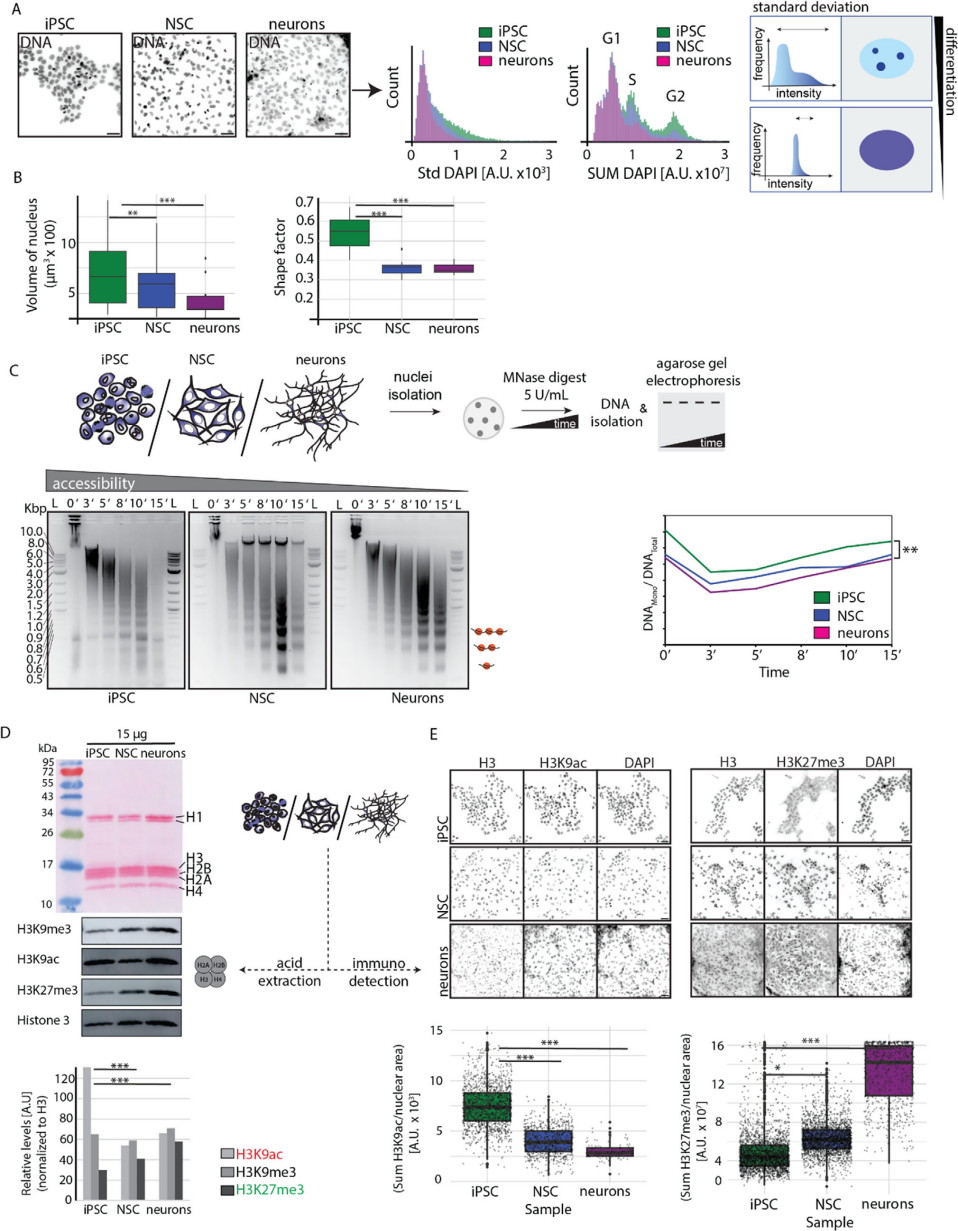
Chromatin accessibility and modifications during differentiation. A) Total DNA was detected using DAPI and histograms depicting the standard deviation (std DAPI) and total DNA amount (DAPI SUM) distribution and diversity of cell populations for each cell differentiation state. Scale bars: 50 µm. B) Box plots quantifying the changes in the volume of the nucleus (µm^3^) and shape factor properties of the cell differentiation states as indicated (3 biological replicates, p values: ^***^(*p* ≤ 0.001) and ^**^(*p* ≤ 0.01)). C) Genome‐wide chromatin accessibility profiles using the MNase digestion assay (5 U mL^−1^) over time. The line plot shows the DNA mono/DNA total (y‐axis) and time (x‐axis) in minutes (2 biological replicates, p values: ^**^(*p* ≤ 0.01)). D) The histone fractions were isolated from different cell differentiation states using the acid extraction method and western blots were used to detect the different histone modifications. The bar plots show the relative histone modification levels normalized to the total histone H3 level (2 biological replicates, p values: ^***^, *p* ≤ 0.001). E) Representative images show different histone modifications, total histone H3 and DNA (DAPI) using immunostaining. The data was analyzed and represented as a boxplot for different modifications normalized to histone H3 and nuclear area. The representative microscopy images are shown in gray scale (2 biological replicates, p values: ^***^(*p* ≤ 0.001) and ^*^(*p* ≤ 0.05)). Scale bars: 50 µm.

To analyze the DNA distribution of different cell types, we stained the DNA of iPSCs, NSCs, and neurons using DAPI. High‐throughput imaging was then employed to quantify the standard deviation (Std DAPI) and total DNA amounts (SUM DAPI), which are shown in **Figure** **3**A. Briefly, the nuclei were first segmented using Knime analytics software, and the total DNA intensities (SUM DAPI) and standard deviation of DAPI (Std DAPI) measurements per cell were obtained. A higher standard deviation indicates greater variability in DNA compaction (euchromatin versus heterochromatin) across the cell population. Conversely, a lower standard deviation implies an excess of one state of chromatin. Our findings reveal that iPSCs exhibit a greater variation in standard deviation compared to NSCs and neurons, indicating a distribution of both euchromatin and heterochromatin within the iPSC population (Figure 3A). The histogram comparing total DNA amounts (SUM DAPI) across the cell types displays that iPSCs (green) include populations in the G1, S, and G2 phases, whereas NSCs contain a lower number of cells in the G2 phase relative to iPSCs. Notably, neurons predominantly consist of G1 phase cells (Figure 3A). Additionally, we obtained 3D stacks of the DNA dye from all cell types and measured the volume (in µm^3^) and shape factor, presenting the results as boxplots in Figure 3B. During neuronal differentiation, both NSCs and neurons significantly decrease in volume compared to iPSCs and exhibit increased irregularity in shape (Figure 3B).

We, then, examined whether the observed changes in global chromatin reorganization affect chromatin accessibility. To test this hypothesis, we performed a micrococcal nuclease (MNase) digestion assay to assess global chromatin accessibility in iPSCs, NSCs, and neurons. Briefly, we applied 5 U mL^−1^ MNase for various time intervals (0, 3, 5, 8, 10, and 15 min) to isolated genomic DNA. Subsequent agarose gel electrophoresis analysis revealed that, over time, the MNase‐digested genomic DNA (detected by ethidium bromide staining) progressively transitioned from larger fragments, indicative of multiple nucleosomes, toward single nucleosome‐sized fragments, observed between 10 and 15 min of digestion (Figure 3C). Next, we quantified the ratio of intensities of DNA fragments under 0.5 kbp (DNAmono) and total DNA (DNAtotal) within the same gel lane. The plot of the DNAmono/DNAtotal ratio (y‐axis) against time (x‐axis) revealed that chromatin accessibility was significantly higher in iPSCs compared to NSCs and neurons (Figure 3C). Interestingly, we found no significant differences in chromatin accessibility between NSCs and neurons (Figure 3C).

To investigate whether changes in histone modifications during neuronal differentiation affect global chromatin profiles and accessibility, we extracted histones from iPSC, NSC, and neuron cell pellets using established acid extraction protocols. A representative image of a gel analysis, visualized by Ponceau staining, revealed the presence of extracted proteins, including the different histones (H1, H2A, H2B, H3, H4) (Figure 3D). We then detected by western blot analysis prominent histone modifications associated with different chromatin states using specific antibodies (Figure 3D). These included H3K9me3 (constitutive heterochromatin mark), H3K9ac (euchromatin mark), and H3K27me3 (facultative heterochromatin mark). Canonical histone H3 was also detected, and its levels served as a loading control for normalization. The intensity of each histone modification band was normalized to the corresponding H3 band intensity, and the relative signal levels were plotted as bar graphs using Image Lab software (Figure 3D). The western blot results indicated that global levels of H3K9ac decreased, while H3K27me3 levels increased (Figure 3D). Notably, the levels of H3K9me3 did not change significantly. To further validate these findings, we performed immunofluorescence staining for H3K9ac and H3K27me3, along with total histone H3 (Figure 3E). Representative grayscale images illustrate the localization of these histone modifications and DNA staining within the cells (Figure 3E). Quantification of the fluorescence intensity in individual nuclei was performed using Knime analytics software and presented as boxplots (Figure 3E; Table S9, Supporting Information), which corroborated the western blot results. During neuronal differentiation, H3K9ac levels decreased while H3K27me3 levels increased. iPSCs showed high H3K9ac levels, indicating active chromatin, which aligns with findings in mouse pluripotent stem cells.^[^ ^69^ ^]^ The co‐occurrence of H3K4me3 and H3K27me3 marks in iPSC populations has been associated with “poised” chromatin states that support stem cell pluripotency, though our population‐level analysis cannot distinguish between true bivalency at individual loci versus cellular heterogeneity.^[^ ^70^ ^]^ iPSCs high proliferation rate leads to many cells in S phase with shorter G1 phase ​​.^[^ ^71^, ^72^ ^]^ However, slower H3K27me3 restoration after replication results in diluted marks.^[^ ^73^ ^]^ As cells differentiate, iPSCs' hyperactive chromatin state reduces, with lower H3K9ac levels. The poised chromatin becomes silenced through H3K27me3 deposition. More cells enter G1 phase (NSCs) or stop replicating (neurons), creating a transcriptionally repressed chromatin state with increased H3K27me3 marks, demonstrated by reduced chromatin accessibility to MNase digestion (Figure 3C) and altered DNA intensity distributions (Figure 3A) in differentiated states. The increased H3K27me3 levels may mechanistically contribute to reduced mobility through H1‐mediated nucleosome compaction and PRC2 feedback loops, while decreased H3K9ac may reduce chromatin accessibility.^[^ ^74^, ^75^ ^]^ To quantitatively define chromatin structural changes during differentiation, we measured chromatin accessibility using MNase digestion assays, which revealed significantly reduced nuclease sensitivity in NSCs and neurons compared to iPSCs (Figure 3C). We define “chromatin compaction” here as this measurable reduction in accessibility to enzymatic digestion, rather than overall chromatin volume changes. Similarly, we define “reduced plasticity” as the observed decrease in chromatin mobility (measured by mean square displacement analysis) combined with increased repressive histone modifications that correlate with reduced transcriptional competence.

### 2.4 Chromatin Motion is Confined During Neural Differentiation

**Figure 4:**
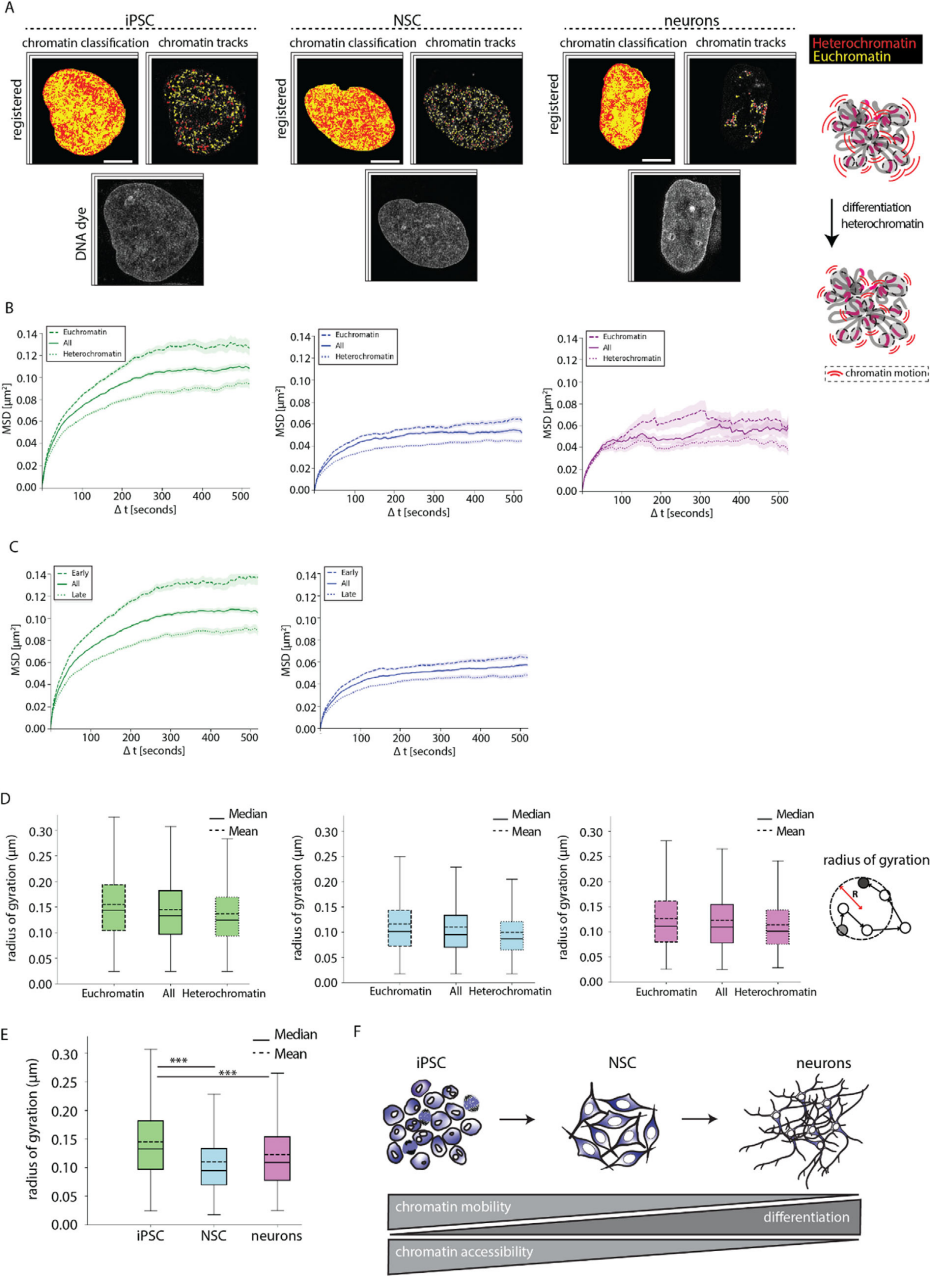
Correlation of chromatin mobility and chromatin states. A) The live‐cell DNA dye was used to classify DNA into euchromatin and heterochromatin and the corresponding tracks of dC^SiR^TP labeled chromatin into two states – euchromatin (yellow) and heterochromatin (red). B) The tracks within the chromatin states were further classified and used to perform mean square displacement analysis. The MSD (µm^2^) over time (Δt, seconds) was plotted for euchromatin, heterochromatin and combined. C) The chromatin tracks within the cells were further classified into early and late categories based on nucleotide label and used to perform mean square displacement analysis. The MSD (µm^2^) over time (Δt, seconds) was plotted for early, late, and all (combined). D) Radius of gyration (µm) for euchromatin, combined (all), and heterochromatin chromatin tracks as boxplots with mean and median values for iPSCs, NSCs, neurons. E) Comparison of radius of gyration (all) for iPSCs, NSCs, neurons as boxplots. F) Graphical illustration of the relationship between chromatin mobility, accessibility and neuronal differentiation. Scale bars: 5 µm.

To investigate whether the labeled chromatin sites exhibit different diffusion or mobility based on their chromatin state, we classified total DNA into two major categories based on pixel intensity (**Figure** **4**A; heterochromatin–red, euchromatin–yellow) in live‐cell imaging using a live‐cell DNA dye. We then applied this classification as a mask to categorize the labeled tracks into corresponding euchromatin or heterochromatin tracks. This allowed us to track and obtain the MSD of labeled tracks located within either euchromatin or heterochromatin regions of the nucleus (Figure 4B). Consistent with previous reports,^[^ ^21^, ^22^ ^]^ we observed that chromatin labeled as heterochromatin is less mobile than chromatin labeled as euchromatin. As we observed in the histone modifications experiments, the decrease in the euchromatin mark (H3K9ac) during neuronal differentiation suggests that the reduced mobility of chromatin foci in heterochromatin regions contributes to the overall decrease in chromatin mobility observed in NSCs and neurons, as compared to iPSCs (see Figure 2G,H). However, the relative difference in mobility between heterochromatin tracks and the combined pool of all tracks was not greater in NSCs and neurons compared to iPSCs. Earlier studies have also shown that chromatin changes are initiated by neural pioneer transcription factors (Sox4, Sox11, Pax6, NeuroD1) that directly access nucleosomal DNA and recruit chromatin remodelers, integrating developmental signaling pathways to shift from accessibility‐promoting to compaction‐favoring activities.^[^ ^76^, ^77^ ^]^

Additionally, we have computed the radius of gyration (µm), which essentially quantifies how much nuclear space the chromatin can explore within a given time. We plotted the radius of gyration (µm) for 500 s of imaging time for euchromatin, combined (all), and heterochromatin chromatin tracks for iPSCs, NSCs, neurons, and observed that overall heterochromatin is more constrained than euchromatin or both combined (Figure 4C). We then compared the radius of gyration (µm) for 500 s of imaging time for iPSCs, NSCs, and neurons and observed that the iPSCs have a larger radius of gyration (µm) than NSCs and neurons, allowing us to conclude that the chromatin in the neural differentiation state is more constrained than in the pluripotent state (Figure 4D). We propose that the restriction in chromatin accessibility during differentiation is the main cause for the reduction of chromatin mobility (Figure 4E).

In conclusion, we have developed a novel STED‐compatible method for labeling genomic DNA in living human induced pluripotent stem cells (iPSCs) and neural stem cells (NSCs). Our study contributes to the growing understanding of chromatin dynamics during differentiation by providing direct DNA labeling rather than (single) protein‐based measurements, quantifying chromatin mobility at a super‐resolution scale during human neural differentiation. While the general concept of differentiation‐associated mobility changes has been established across different cell types, our approach offers novel mechanistic insights into the molecular basis of these changes during human neurogenesis.

Our method utilizes a novel silicon rhodamine‐linked deoxycytidine triphosphate (dCSiRTP), which is delivered to live‐cells by the SNTT1 transporter. The dCSiRTP/SNTT1 combination enabled rapid chromatin labeling (within 10–15 min) without disrupting cell morphology, allowing for immediate high‐resolution imaging and for unimpaired cell proliferation and differentiation. This approach is straightforward and highly efficient, offering significant advantages over previously reported methods, including our own.^[^ ^21^, ^32^, ^33^ ^]^ Using this system, we achieved high‐resolution quantification and tracking of chromatin dynamics in living cells throughout the differentiation process from iPSCs to NSCs and ultimately to neurons. Our analyses reveal a progressive decrease in chromatin mobility that correlates with cellular differentiation state, strongly suggesting a relationship between genome dynamics and developmental potential. As NSCs are still proliferating but show similar reduced chromatin mobility to neurons, this effect cannot be explained by cell cycle differences alone. Our observation of reduced chromatin mobility during cellular differentiation is consistent with recent studies that demonstrated, using fluorescent recovery after photobleaching (FRAP), that chromatin mobility serves as a readout of the cellular differentiation state and reflects the underlying changes in chromatin organization.^[^ ^78^ ^]^ The reduced chromatin mobility observed during neural differentiation (from 14.99 to 8.38 × 10^−5^ µm^2^ s^−1^) may reflect the establishment of stable transcriptional programs essential for neuronal function, correlated with reduced chromatin accessibility (as measured by MNase sensitivity) and increased repressive histone modifications (H3K27me3), providing a physical basis for cellular memory and identity maintenance.

Our quantitative measurements of chromatin mobility provide new mechanistic insights into how chromatin organization changes during differentiation. The progressive reduction in chromatin mobility we observe (from 14.99 to 8.38 × 10^−5^ µm^2^ s^−1^) suggests that restricted movement may be mechanistically coupled to differentiation rather than merely correlative. Reduced mobility may serve to enforce irreversible cell fate decisions by creating “chromatin state barriers” that restrict access to pluripotency sites^[^ ^79^ ^]^ could actively contribute to cell fate commitment by constraining enhancer‐promoter search processes, limiting transcriptional noise, and stabilizing cell type‐specific gene expression programs through restricted chromatin accessibility.^[^ ^80^ ^]^ This mobility‐accessibility relationship provides a foundation for understanding how the physical properties of chromatin domains facilitate the transition from pluripotent to differentiated states, opening new avenues for investigating the mechanistic role of chromatin dynamics in cell fate decisions. Our labeled chromatin domains likely encompass multiple levels of chromatin organization, spanning from tens of kilobases to megabases of DNA, i.e., from chromatin loop‐size to TAD‐size structures. The mobility changes we observe may reflect reorganization at multiple scales: from nucleosome‐level compaction to higher‐order folding within and between chromosomal domains, providing a complementary perspective to Hi‐C‐based structural studies. Our findings align with recent work demonstrating that transcription‐coupled changes in chromatin mobility^[^ ^81^, ^82^ ^]^ and replication‐dependent labeling approaches^[^ ^83^ ^]^ yield insights into the physical properties governing chromatin organization during cellular differentiation.

## 3 Experimental Section

### Synthesis of dC^SiR^TP

dC^SiR^TP was prepared by reaction of azido‐propargyloxy‐triethylene glycol conjugated deoxycytidine triphosphate dC^pegN3^TP with commercially available bicyclononyne‐linked silicon rhodamine fluorophore SiR‐BCN in dimethylsulfoxide at room temperature. The product was isolated in 50% yield after purification by reverse‐phase HPLC. Experimental conditions for the reaction, analytical data of the product (^1^H and ^13^C NMR, MS), photophysical characterization data, as well as description of the biochemical experiments (in vitro DNA incorporation: PEX, PCR) are provided in Tables S1–S3 and Figures S1–S7 (Supporting Information).

### Cells

All cells used were tested for mycoplasma and deemed free of contamination. All cell lines were authenticated by STR profiling (ATCC). All cells were grown in a humidified atmosphere of 5% CO2 at 37 °C. The details of the cell lines used were described in Table S4 (Supporting Information). To grow the human induced pluripotent stem cells clone B4 (hiPSC),^[^ ^84^ ^]^ surfaces were first coated with vitronectin (VTN‐N) recombinant human protein (VTN) 5 ng mL^−1^ (Cat.No.: A14700, Thermo Fisher Scientific, USA) for 1 h. The iPSCs clone B4 were grown in iPSC growth media containing IPS‐Brew basal medium (Cat.No: 130‐107‐086, Miltenyi Biotec, Germany) supplemented with IPS‐Brew 50x supplement (Cat.No: 130‐107‐087, Miltenyi Biotec, Germany) and Revita Cell supplement (1x) (Cat.No: A2644501, Fisher Scientific, USA) without antibiotics. For subculturing of iPSCs, the cells were detached using 1x accutase (Cat.No: A6964, Sigma Aldrich Chemie, Germany) at 37 °C for 3 min, followed by centrifugation at 300 x g for 3 min. The excess media was then removed, and cells were resuspended in the growth media and seeded on vitronectin (VTN‐N) coated dishes. All iPSCs cells were grown till they started forming colonies before performing experiments (Figure S8, Supporting Information). The iPSCs were differentiated in vitro to neural stem cells (NSCs) (Figure S9, Supporting Information), and the detailed protocol and media compositions are described in the Section S6 and Table S7 (Supporting Information).

### Immunofluorescence

Cells on coverslips were fixed with 3.7% formaldehyde for 10 min. Cells were permeabilized with 1x PBS supplemented with 0.7% Triton X‐100 for 20 min. After washing three times with PBS‐T for 5 min, cells were blocked with 1% BSA/PBS for 30 min and subsequently incubated for an hour with the primary antibody, followed by three washing steps with PBS‐T. Cells were incubated with a secondary antibody for 45 min, followed by three washing steps with PBS‐T. DNA was counterstained with 4′,6‐diamidino‐2‐phenylindole (DAPI) (Cat. No. 6335.1, Carl Roth, Germany) for 10 min. Coverslip was mounted onto glass slides using Vectashield before proceeding to imaging.

iPSCs were seeded and cultured on glass coverslips until colonies were obtained. The media was replaced with media containing 10 µM EdU and pulsed for 10 min (Table S5, Supporting Information). Subsequently, the cells were fixed with 3.7% formaldehyde for 10 min. The samples were used to detect pluripotent stem cell markers (Sox2, Oct3/4), and EdU was detected using Click‐IT chemistry (Table S8 and Figure S8, Supporting Information).

### Micrococcal Nuclease Chromatin Accessibility Assay

A total of 10 × 10^6^ cells were harvested, and cell pellets were resuspended in a hypotonic lysis buffer (10 mM Tris, pH 7.5, 10 mm NaCl, 3 mm MgCl_2,_ and 0.5% NP‐40) for 5 min on ice. Cell nuclei were pelleted (5 min, 120 × g), washed once in digestion buffer (50 mm Tris, pH 8, 5 mm CaCl_2_), and resuspended in digestion buffer. Microccocal nuclease (MNase) (cat# EN0181, Thermo Fisher Scientific, Waltham, MA, USA) was diluted in MNase storage buffer (20 mm TrispH7.6, 50 mm NaCl, 50% glycerol), 5 U mL^−1^ and added to the nuclei in a final volume of 100 µL. Reactions were incubated for different time points at 37 °C, stopped by the addition of stopping buffer (20 mm EDTA, 0.4% SDS, 0.5 mg mL^−1^ proteinase K), and incubated at 50 °C overnight. DNA was recovered by adding 0.1 volumes of 3 M NaCl and 2 volumes of 100% ethanol and storing at −20 °C for an hour. After centrifugation (15 min, 15 000 × g) and washing with 70% ethanol, the DNA pellet was air‐dried and resuspended in 50 µL ddH_2_O. Equal amounts of DNA were loaded on a 1.5% agarose gel and electrophoresed for 45 min at 100 V. Gels were stained with ethidium bromide in 1× TAE buffer (Tris‐acetate‐EDTA) and imaged using an AI600 imager. Integrated intensities of monomeric DNA and total DNA in each lane were measured with ImageJ (Table S9, Supporting Information) and plotted as a DNA monomer/DNA total ratio.

### Western Blot

The cells were cultured as described above. The cells were detached using 2 mL of accutase (Cat.No: A6964, Sigma–Aldrich Chemie, Germany) and counted, and 2 × 10^6^ cells were collected into a tube. The lysis buffer NP‐40 was supplemented with the protease inhibitors: 1 mM PMSF (phenylmethylsulfonyl fluoride, Cat.No: 6367.2, Carl‐Roth, Germany), 1 mM AEBSF (4‐(2‐Aminoethyl) benzyl sulfonyl fluoride hydrochloride, Cat.No: A1421.0100, VWR, USA), 1 mM E64  (Cat.No: E3132, Sigma‐Aldrich, USA) and 1 nM Pepstatin A (Cat.No: 77170, Sigma–Aldrich, USA). Cells were homogenized in cell lysis buffer containing 150 mM NaCl (Cat.No.: 0601.2, Carl Roth, Karlsruhe, Germany), 200 mM TrisCl pH 8 (Cat.No.: A1086.500, Diagonal, Münster, Germany), 5 mM EDTA (Cat.No.: 8040.2, Carl Roth, Karlsruhe, Germany) 0.5% NP‐40 (Cat.No.: 74385, Sigma–Aldrich Chemie GmbH, Steinheim, Germany and incubated for 30 min at 4 °C. The lysates were cleared by centrifuging for 30 min at 14 000 g at 4 °C. Protein concentration was determined using Pierce 660 nm Protein Assay Reagent (cat# 22660, Thermo Fisher Scientific, Waltham, MA, USA) according to the manufacturer's instructions. Protein lysates were denatured in 6x protein loading dye (560 mM Tris‐HCl, pH 6.8, 60 mM DTT, 6 mM EDTA, 30% glycerol, and 0.6% bromophenol blue) for 5 min at 95 °C. Denatured protein lysates were separated by 10% SDS‐PAGE and transferred onto 0.45 µm nitrocellulose membranes (GE Healthcare/Whatman; Catalog #10600002). The membranes were blocked with 3% low‐fat milk for 30 min and subsequently incubated with primary antibodies diluted in blocking solution for 2 h at 4 °C (Table S8, Supporting Information). After washing three times with PBS‐T for 5 min each, membranes were incubated with secondary antibodies for 1 h at room temperature (Table S8, Supporting Information). Following two wash steps with PBS‐T, protein bands on the membranes were visualized using an AI600 imager (GE Healthcare) (Table S6, Supporting Information).

### Acid Extraction of Histones

The histone proteins were acid‐extracted using the protocol described in.^[^ ^85^ ^]^ ≈10^6^ cells were taken from each cell type and centrifuged. The cells were washed with 1x PBS before resuspending in 1 mL of hypotonic lysis buffer (10 mm Tris‐Cl pH 8.0, 1 mm KCl, 1.5 mm MgCl_2_, and 1 mm DTT. Protease inhibitors were supplemented before use). The cells were incubated for 30 min on a rotator for hypotonic swelling and lysed by mechanical shearing. The nuclei were pelleted using centrifugation at 10 000 x g for 10 min at 4 °C. The nuclei were resuspended in 0.4 N H_2_SO_4_ and incubated overnight. The cells were then carefully resuspended using 200 µL pipette tips. The nuclei debris was removed by centrifuging the mixture at 16 000 x g at 10 min, and the supernatant was transferred to a new tube. ≈132 µL of 100% TCA (Trichloroacetic acid) was added dropwise to make a final concentration of 33% and inverted several times to precipitate the histones. The samples were incubated on ice for 30 min. The lysate was centrifuged at 16 000 x g for 10 min at 4 °C. The supernatant was removed, and the pellet was washed twice with ice‐cold acetone to remove the acid. After drying the histones at room temperature for 20 min, they were dissolved in 50 µL of ddH_2_O. The concentration of the proteins was measured using Pierce 660 nm Protein Assay Reagent (cat# 22660, Thermo Fisher Scientific, Waltham, MA, USA). Protein lysates were denatured in 6x protein loading dye (560 mm Tris‐HCl pH 6.8, 60 mm DTT, 6 mm EDTA, 30% glycerol and 0.6% bromophenol blue) for 5 min at 95 °C. 10 µg of denatured protein lysates were resolved using 10% SDS‐PAGE and transferred onto 0.45 µm nitrocellulose membranes (GE Healthcare/Whatman; Catalog #10600002). The membranes were blocked with 3% low‐fat milk for 30 min and subsequently incubated with primary antibodies diluted in blocking solution for 2 h at 4 °C (Table S8, Supporting Information). After washing three times with PBS‐T for 5 min each, membranes were incubated with secondary antibodies for 1 h at room temperature (Table S8, Supporting Information). Following two wash steps with PBS‐T, protein bands on the membranes were visualized using an AI600 imager (GE Healthcare) (Table S6, Supporting Information).

### Chromatin Labeling Using SNTT1

The iPSCs and NSCs were cultured as described above. The cells were split onto glass bottom culture dishes for chromatin labeling and incubated with tricine buffer (pH 7.4) containing 10 µm SNTT1 (Cat.No: SCT064, Merck Millipore, Germany) + 10 µm dC^SiR^TP for 15 min at 37 °C (Table S5, Supporting Information). The cells were then washed with 1 x PBS to remove excess nucleotides. The cells were subsequently incubated in media containing 10 µm of Abberior DNA live 590 dye (Cat.No: LV590‐0143‐50UG, Abberior, Germany) or Abberior tubulin live 610 dye (Cat.No: LV610‐0141‐50UG, Abberior, Germany) for 3 h (Table S5, Supporting Information).

### Chromatin Compaction Analysis of Replication Labeling

IPSC cells were labeled with nucleotides using scratch loading on coverslips. The next day, the cells were fixed using 3.7% formaldehyde for 10 min. Then, 1 mg mL^−1^ 4′,6‐diamidino‐2‐phenylindole (DAPI) (Cat. No: 6335.1, Carl Roth, Germany) was used to stain the DNA for 10 min. The cells were then imaged in 3D (Z stacks) using super‐resolution 3D‐SIM to image DNA and nucleotides. The reconstructed and thresholded 16‐bit super‐resolved images were analyzed using the “Nucim” library available on the platform “R” to subdivide individual nuclei into chromatin compaction classes and map signals from other channels to individual compaction classes. First, the DAPI channel was segmented and used to mask the region of interest. Individual voxels within a single nucleus were assigned to a certain compaction class based on the probability of this voxel belonging to the same class computed from a hidden Markov random field (HMRF) stochastic model, classifying the nuclei into seven different compaction classes, where the first class represents interchromatin (IC), as opposed to the chromatin compartment of chromatin domain clusters (CDCs). Classes two and three represent less compacted perichromatin located toward the surface of CDCs, which together with IC form the active nuclear compartment (ANC), whereas classes four to seven, located toward the interior of chromatin domain clusters (CDC), represent the more compacted inactive nuclear compartment (INC) (Figure 2A). Spots from other channels were further mapped into these subclasses based on a combined threshold and intensity method, where first the spots were segmented with the threshold method, followed by an intensity‐weighted calculation of the relative fraction, leading to more intense signals having a larger impact and low‐intensity signals having less impact.

### Super‐Resolution Time‐Lapse Microscopy

The chromatin and total DNA were labeled in live‐cells as described above. Following the labeling, the cells on the glass bottom were used to perform live/fixed cell time‐lapse microscopy for chromatin mobility measurements. Briefly, time‐lapse imaging was performed within 1–2 h after labeling of the cells using a Zeiss LSM 900 Airyscan system (Zeiss, Germany) equipped with a C Plan‐ Apochromat 63x oil objective and Axiocam 820 mono SONY IMX541 CMOS camera with Airyscan detector for high‐speed stack acquisition. The details of the microscope are described in Table S6 (Supporting Information). The DNA dye was used to find the focal plane of cells to minimize bleaching in the chromatin (dC^SiR^TP) channel. The DNA dye and dC^SiR^TP were excited using 561 and 640 nm lasers, respectively. All chromatin motion time‐lapse microscopy was performed using two‐channel imaging and 5 z‐stacks per channel with a lateral (x‐y) resolution of 48 nm and axial resolution (z) of 170 nm with 5 s interval. The raw data were processed to obtain super‐resolution time‐lapse movies using Airyscan joint convolution with 20 iterations of processing (Zeiss) (Table S9, Supporting Information). The time‐lapse imaging was performed at 37 °C with a humidified atmosphere using an environmental chamber (Table S6, Supporting Information). The standard protocol for examining chromatin mobility in dC^SiR^TP labeled nuclei proceeded in the following manner: first, a reference overview image of dC^SiR^TP and Abberior DNA live 590 dye using wide‐field mode was collected from a single focal plane corresponding to the middle of the nucleus. Second, while maintaining the same central focal plane, a time series (frame interval of 5 s, 5 z‐stacks, 200 frames) and 3D volume with fewer stacks were captured to minimize photo toxicity. The imaging was performed over multiple experiments to have reproducibility and sufficient replicates.

Fixed cell imaging was performed as an imaging control, where the cells were labeled with chromatin (dC^SiR^TP) and Abberior DNA live 590 dye. Post labeling, the cells were fixed using 3.7% formaldehyde for 10 min at room temperature. The imaging was performed as described above.

For fixed cell STED super‐resolution imaging, the iPSCs were cultured as described above and seeded on high precision (18 × 18 mm) square coverslips. The cells were labeled with chromatin (dC^SiR^TP) and Abberior DNA live 590 dye. The cells were allowed to recover from the labeling for 2–3 h and fixed using 3.7% formaldehyde for 10 min at room temperature. The coverslip was then mounted using Vectashield before proceeding to imaging using STED 775 QUAD Scan microscope (Abberior Instruments) (Table S6, Supporting Information).

### 3D Motion Analysis of Chromatin

To decouple the movement of the chromatin structures from the movement and deformation of the cell, joint affine and non‐rigid 3D image registration was performed using the method in^[^ ^86^ ^]^ adapted for 3D registration. To ensure that image registration does not affect the motion of the chromatin structures, registration was performed using the DNA (DAPI) channel images. The transformation parameters obtained were subsequently applied to the chromatin (labeled nucleotide) channel. Each frame of a live‐cell image sequence was registered to the first frame.

The 3D chromatin structures within cell nuclei were tracked in the registered 3D live‐cell fluorescence microscopy image sequences using a probabilistic particle tracking method.^[^ ^87^ ^]^ The method combines Kalman filtering with particle filtering and uses sequential multi‐sensor data fusion and separate sensor models to integrate multiple measurements. Detection‐based and prediction‐based measurements were obtained by elliptical sampling.^[^ ^88^ ^]^ For detecting 3D chromatin structures (foci), the spot‐enhancing filter (SEF)^[^ ^89^ ^]^ was used, which uses the Laplacian of Gaussian (LoG) filter followed by thresholding and computing local maxima. The threshold was automatically determined for each image sequence as the mean of the LoG filter responses plus the standard deviation times a factor. The factor was chosen so that all relevant foci were detected while detections due to noise were avoided. To investigate whether different foci sizes lead to differences in diffusivity, the median foci size of a trajectory was determined, and the foci were distinguished into small and large by having an even split (same number of trajectories) (Figure S13A–C, Supporting Information).

To quantify the mobility of the 3D chromatin structures, a mean‐square displacement (MSD) analysis was performed.^[^ ^90^ ^]^ For this, the MSD over time intervals Δt was calculated for each trajectory and then averaged. To determine the (short‐range) diffusion coefficient D [µm^2^s^−1^], the diffusion model 6*Dt* + *b* was fitted to the linear part at the beginning (first 50 s) of the averaged MSD curves^[^ ^90^ ^]^ (Figure S13D, Supporting Information). The bias parameter b accounts for localization errors. In addition, the anomalous diffusion model 6Γ*t* ^α^ was fitted to the whole averaged MSD curves (Figure S13E, Supporting Information) yielding the motion type parameter α, where a value of α < 0.9 indicates sub‐diffusion and a decrease in α corresponds to more constrained motion. To improve the robustness of the motility analysis, only trajectories with a minimum time duration of ≈50 s (10 time steps) were considered. To further characterize the 3D motion of the chromatin structures, the radius of gyration^[^ ^91^ ^]^ was computed for each trajectory.

In addition, the mobility of the 3D chromatin structures was analyzed with respect to the 3D nuclear landscape. The 3D cell nucleus was divided into two compaction classes based on the intensity values of the DAPI channel using the method in.^[^ ^92^ ^]^ For each frame of an image sequence, first the cell nucleus was segmented, and then intensity‐based classification using a Gaussian Mixture Model was performed. Computed trajectories were assigned to the compaction class containing the majority of the track points.

### Resolution Gain Analysis of STED Microscopy

The resolution gain between confocal and STED microscopy was quantified using chromatin foci in 2D images of the same cell. For this, the foci were detected in both confocal and STED data using the spot‐enhancing filter (SEF) as described above. The two sets of foci were then matched using the Hungarian method.^[^ ^93^ ^]^ To quantify the resolution of the detected foci, the fluorescence intensity profile was determined along a horizontal line with length 1 µm centered at each detection. The intensity profiles were then averaged over all detections for each imaging method and normalized to the range between 0 and 1, and plotted (Figure 1G). From the normalized averaged intensity profiles, the Full Width at Half Maximum (FWHM) was calculated.

### Statistical Analysis

Statistical analyses were performed using RStudio (Version 2024.12.1 + 563) and KNIME Analytics (version 5.4.2). Data were presented as mean ± standard deviation unless otherwise specified. For comparison between groups, statistical significance was assessed using appropriate tests as follows:

Chromatin mobility analysis: mean square displacement (MSD) curves were fitted using standard diffusion models (6Dt + b for short‐range diffusion and 6Γt^α for anomalous diffusion) with a minimum trajectory duration of 500 s (100‐time steps) for robust analysis.
Chromatin accessibility (MNase assays): two‐way analysis was performed with biological replicates (n = 3), and significance was determined using a *t*‐test (statistical test) with p‐values indicated as ^**^(*p* ≤ 0.01).
Histone modification analysis: Western blot quantification was normalized to histone H3 loading controls, with biological replicates (n = 3). Immunofluorescence quantification was performed on individual nuclei using KNIME analytics software with significance levels indicated as ^***^(*p* ≤ 0.001), ^**^(*p* ≤ 0.01), and ^*^(*p* ≤ 0.05).
Nuclear morphology analysis: volume and shape factor measurements were analyzed across biological replicates (n = 3), and significance was determined using a *t*‐test (statistical test) with significance indicated as ^***^(*p* ≤ 0.001) and ^**^(*p* ≤ 0.01).
Radius of gyration analysis: statistical comparison between cell types was performed using median and mean values with significance levels of ^***^(*p* ≤ 0.001).

All experiments included appropriate biological replicates as indicated in figure legends, and statistical significance was set at *p* < 0.05 unless otherwise specified.

## Conflict of Interest

The authors declare no conflict of interest.

## Author Contributions

M.K.P. performed cell culture, differentiation, chromatin labeling, time‐lapse microscopy, chromatin condensation, and respective analyses. M.K. synthesized and characterized the dC^SiR^TP, T. K. performed initial experiments on DNA labeling of somatic cell types using the dC^SiR^TP / SNTT1 method and contributed to writing of the manuscript, K.C. and J.M. performed image motion analysis, S.K.P. performed histone modification experiments and analysis, A.M. and H.H. performed STED imaging, H.L., K.R., M.H., and M.C.C. supervised the study and acquired funding. M.K.P. wrote the manuscript draft and figures with input from all authors.

## Supporting information



Supporting Information

Supplemental Movie 1

Supplemental Movie 2

Supplemental Movie 3

Supplemental Movie 4

## Data Availability

All data are available at the TU Data lib at (https://doi.org/10.48328/tudatalib‐1651). Renewable biological materials and software will be made available upon request from M. Cristina Cardoso (cardoso@bio.tu‐darmstadt.de), Michal Hocek (hocek@uochb.cas.cz) and Karl Rohr (k.rohr@dkfz.de). All experimental data including mass spectrometry and NMR spectra are presented in supplementary files.
