# Supplementary material for: Super‐Resolution Compatible DNA Labeling Technique Reveals Chromatin Mobility and Organization Changes During Differentiation
Source: Adv Sci (Weinh). 2025 Sep 9;12(45):e05955. doi: 10.1002/advs.202505955 (PMC12677680; doi:10.1002/advs.202505955)
Supplement: Supplementary file 1 — Supporting Information [file ADVS-12-e05955-s005.docx]

**SUPER-RESOLUTION COMPATIBLE DNA LABELING TECHNIQUE REVEALS CHROMATIN MOBILITY AND ORGANIZATION CHANGES DURING DIFFERENTIATION**

Maruthi K. Pabba^1^, Miroslav Kuba^2,3^, Tomáš Kraus^2^, Kerem Celikay^4^, Janis Meyer^4^, Sunil Kumar Pradhan^1^, Andreas Maiser^5^, Hartmann Harz^5^, Heinrich Leonhardt^5^, Karl Rohr^4^, Michal Hocek^2,3*^, M. Cristina Cardoso^1*^.

1. Cell Biology and Epigenetics, Department of Biology, Technical University of Darmstadt, 64287 Darmstadt, Germany
2. Institute of Organic Chemistry and Biochemistry, Czech Academy of Sciences, CZ-16000 Prague 6, Czech Republic.
3. Department of Organic Chemistry, Faculty of Science, Charles University, CZ-12843 Prague 2, Czech Republic
4. Biomedical Computer Vision Group, BioQuant, IPMB, Heidelberg University, Germany
5. Human Biology and Bioimaging, Faculty of Biology, Ludwig Maximilians University Munich, 81377 Munich, Germany

* Corresponding authors:

Michal Hocek. Tel: +420 220 183 324; Email:[hocek@uochb.cas.cz](mailto:hocek@uochb.cas.cz)

M. Cristina Cardoso. Tel: +49 6151 16 21882; Email: [cardoso@bio.tu-darmstadt.de](mailto:cardoso@bio.tu-darmstadt.de)

# Supplementary information

## Contents page

1. Synthesis

1.1. General remarks

1.2. Silicon rhodamine modified deoxycytidine-5’-*O*-triphosphate (dC^SiR^TP)

2. Photophysical properties

3. Biochemistry

3.1. Enzymatic synthesis of modified DNA bearing one silicon rhodamine modification by primer extension (gel analysis)

3.2. Preparation of modified dsDNA bearing one silicon rhodamine modification by primer extension (semi-preparative scale)

3.3. Enzymatic incorporation of dC^SiR^TP by polymerase chain reaction

4. Copy of ^1^H, ^13^C and ^31^P NMR spectra (dC^SiR^TP)

5. Copies of mass spectra

6. Generation of neural stem cells and differentiation

7. Supplementary movies

##

## 1. Synthesis

### 1.1. General remarks

Solvents and reagents were purchased from commercial suppliers (Sigma-Aldrich, AlfaAesar, Spirochrome). The reactions were monitored by thin-layer chromatography (TLC) using Merck silica gel 60 F254 plates and visualized by UV (254 nm). Purification by column chromatography was performed using silica gel (40–63 µm). Separations of dC^SiR^TP were performed using HPLC (Waters modular HPLC system) on a column packed with 10 μm C18 reversed phase (Phenomenex, Luna C18). NMR spectra were measured on Bruker AVANCE 500 (^1^H at 500.0 MHz, ^13^C at 125.7 MHz, ^31^P at 202.3 MHz,) NMR spectrometer in D_2_O at 25 °C. Chemical shifts (in ppm, δ scale) were referenced to *t*BuOH = 1.24 ppm. Coupling constants (*J*) are given in Hz, chemical shifts in ppm (δ scale). Complete assignment of all NMR signals was achieved by using a combination of H,H-COSY, H,C-HSQC and H,C-HMBC experiments. Mass spectra and high-resolution mass spectra were measured by ESI ionization technique and spectra were measured on a LTQ Orbitrap XL spectrometer (ThermoFisher Scientific). dC^pegN3^TP was prepared according to a published procedure [^[1]^](https://sciwheel.com/work/citation?ids=16862167&pre=&suf=&sa=0).

### 1.2. Silicon rhodamine modified deoxycytidine-5’-*O*-triphosphate (dC^SiR^TP)

A solution of SiR-BCN (2.5 mg, 3.23 µmol, in 420 µl DMSO) was added to a solution of dC^pegN3^TP (3.5 mg, 3.23 µmol, in 140 µl HPLC grade H_2_O) and the reaction mixture was stirred overnight at room temperature. Then, the DMSO was removed by lyophilization, and the reaction mixture was purified by reverse-phase HPLC (eluent: H_2_O/0.1 M triethylammonium acetate/acetonitrile 37/20/43). Collected fractions were lyophilized, and the excess of the buffer was removed by repetitive freeze-drying from water. The product was obtained as a pale yellow solid (2.84 mg, 50%).

^1^H NMR (500.0 MHz, D_2_O, ref(*t*BuOH) = 1.24 ppm): 0.46, 0.58 (2 × s, 2 × 3H, CH_3_Si); 0.66 – 0.78 (bm, 2H, H-5a,6a-CPCOT); 0.86 (bm, 1H, H-6-CPCOT); 1.10 – 1.24 (bm, 2H, H-5b,7b-CPCOT); 1.27 (t, 27H, *J*_vic_ = 7.3, CH_3_CH_2_N); 1.91, 2.02 (2 × bm, 2 × 1H, H-5a,7a-CPCOT); 2.18, 2.38 (2 × bm, 2 × 1H, H-2ʹ); 2.54 – 2.66 (bm, 1H, H-4b,8b-CPCOT); 2.69 – 2.83 (bm, 1H, H-4a,8a-CPCOT); 2.95 – 3.15 (bm, 2H, NCH_2_CH_2_O); 3.15 – 3.30 (bm, 30H, (CH_3_)_2_N, CH_3_CH_2_N); 3.40 – 3.56, 3.56 – 3.71, 3.71 – 3.77, 3.77 – 3.85, 3.92 (5 × bm, 22H, OCH_2_CH_2_O, NCH_2_CH_2_O, triazole NCH_2_CH_2_O, CH_2_O); 4.09 – 4.23 (bm, 3H, H-4ʹ,5ʹ); 4.30 – 4.41 (bm, 4H, CH_2_C≡C, triazole NCH_2_CH_2_O); 4.55 (bm, 1H, H-3ʹ); 6.12 (bm, 1H, H-1ʹ); 6.49, 6.60 (2 × bm, 2 × 1H, H-2,8-DHBS); 6.90 – 7.04 (bm, 2H, H-1,9-DHBS); 7.20 – 7.29 (bm, 2H, H-4,6-DHBS); 7.69 (bm, 1H, H-3-Ph); 7.95 – 8.03 (H-5,6-Ph); 8.05 (bm, 1H, H-6).

^13^C NMR (125.7 MHz, D_2_O, ref(*t*BuOH) = 30.29 ppm): -2.79, -0.31, -0.28 (CH_3_Si); 8.92 (CH_3_CH_2_N); 17.65, 17.69 (CH-6-CPCOT); 19.28, 19.36, 19.78, 19.81 (CH-5a,6a-CPCOT); 21.79, 22.23 (CH_2_-5,7-CPCOT); 23.04, 25.87 (CH_2_-4,8-CPCOT); 40.14 (CH_2_-2ʹ); 40.50 (NCH_2_CH_2_O); 40.70, 40.75, 40.79 ((CH_3_)_2_N); 40.84 (NCH_2_CH_2_O); 47.36 (CH_3_CH_2_N); 48.46 (triazole-NCH_2_CH_2_O); 59.19 (CH_2_C≡C); 63.80 (CH_2_O); 65.90 (CH_2_-5ʹ); 69.45, 69.65, 69.79, 70.07, 70.22, 70.78 (OCH_2_CH_2_O, triazole-NCH_2_CH_2_O, NCH_2_CH_2_O); 71.21 (CH-3ʹ); 77.61 (CH_2_C≡C); 86.27 (d, *J*_C,P_ = 7.3, CH-4ʹ); 87.10 (CH-1ʹ); 92.40 (C-5, CH_2_C≡C); 114.28, 114.42, 114.49 (CH-2,8-DHBS); 121.02, 121.07, 121.16 (CH-4,6-DHBS); 128.12 (CH-5-Ph or CH-6-Ph); 128.58 (C-9a,10a-DHBS); 129.28 (CH-3-Ph); 129.78 (CH-5-Ph or CH-6-Ph); 135.64 (C-4-Ph); 140.21, 140.25, 140.53 (CH-1,9-DHBS); 140.63 (C-1-Ph); 145.69 (CH-6); 147.64, 147.67, 147.79 (C-4a,5a-DHBS); 153.85, 153.95 (C-3,7-DHBS); 155.95 (C-2); 158.69 (OCONH); 165.20 (C-4); 169.88 (CONH-4-Ph); 173.66 (COO-1-Ph); C-2-Ph, C-10-DHBS and C-3a,8a-CPCOT not detected.

^31^P{^1^H} NMR (202.4 MHz, D_2_O): -22.52, -10.90, -10.18 (3 × bm).

HRMS (ESI^-^): calculated for C_62_H_82_O_23_N_10_P_3_Si: 1455.4542; found 1455.4529.

## 2. Photophysical properties

### Molar absorption coefficients

Absorption coefficients were measured using 1 mL quartz cuvettes.

The absorption coefficients were calculated using the following equation

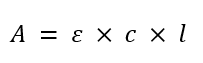


where *A* is the absorbance of the sample, *ε* is the absorption coefficient, *c* is the exact concentration of the sample and *l* is the length of the path that the light travels through the cuvette.

### Fluorescence quantum yields

Relative determination of the fluorescence quantum yields (Ф) was performed using Cresyl violet perchlorate in MeOH (Ф = 0.54) as a reference. The absorbance of sample solutions was kept below 0.10 to avoid inner filter effects. The quantum yields were calculated using the following equation [^[2]^](https://sciwheel.com/work/citation?ids=5424300&pre=&suf=&sa=0).


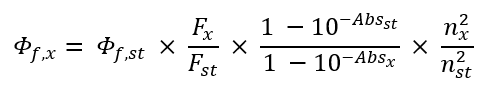


where Φ_f_ is the quantum yield, F is the integrated fluorescence intensity, Abs is the absorbance of the solution at the excitation wavelength, n is the refractive index of the solvent, and the subscripts x and st stand for the sample and standard respectively. Samples were excited at 600 nm and the emission spectra were recorded in the range of 620-800 nm.

## Table S1. Photophysical properties of dC^SiR^TP and DNA19_1C^SiR^.

| **Sample** | **Solvent** | **λ_abs_^a^ (nm)** | **ε^b^ (M^-1^ cm^-1^)** | **λ_em_^c^ (nm)** | **Φ^d^ (%)** |
| --- | --- | --- | --- | --- | --- |
| **dC^SiR^TP** | PBS | 651 | 124800 | 672 | 0.54 |
| **DNA19_1C^SiR^** | PBS | 651 | n.d. | 672 | 0.48 |

*^a^* Position of the absorption maximum, *^b^* molar extinction coefficients, *^c^* position of the emission maximum, *^d^* fluorescence quantum yield measured using Cresyl violet perchlorate in MeOH (Ф = 0.54) as reference, n.d. = not determined.


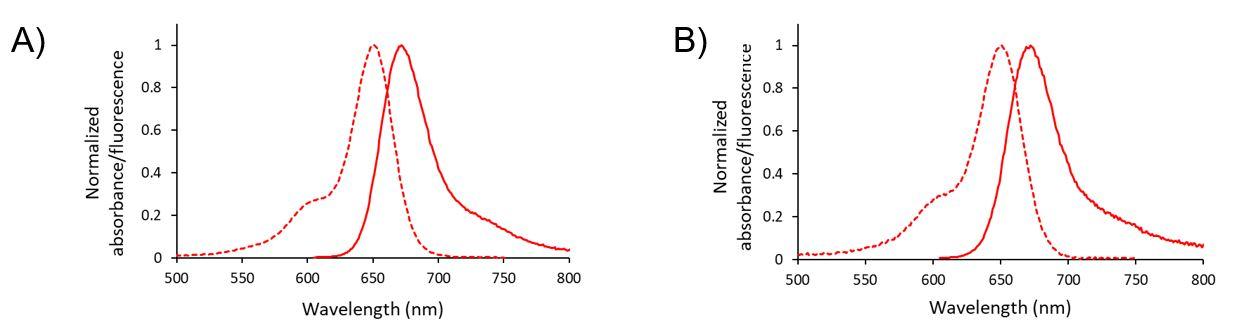


**Figure S1.** Absorption and fluorescence spectrum of **dC^SiR^TP (A)** and **DNA19_1C^SiR^ (B)** in PBS.

## 3. Biochemistry

### 3.1. Enzymatic synthesis of modified DNA bearing one silicon rhodamine modification by primer extension (gel analysis)

The reaction mixture (20 μl) contained FAM labeled primer Prim^PEX^-FAM (for sequence see Table S2; 3 μM, 1 μL), template Temp^PEX^ (for sequence see Table S2; 3 μM, 1.5 μL), KOD XL DNA polymerase (0.25 U/µl, 0.3 μL), natural dGTP (4 mM, 0.6 μL), either natural dCTP (4 mM, 0.3 μL) or dC^SiR^TP (4 mM, 0.3 μL) in corresponding reaction buffer (10x, 2 μL) supplied by the manufacturer. The reaction mixture was incubated for 30 min at 60°C in the thermal cycler. The reaction was stopped by the addition of PAGE stop solution (20 µl) and the reaction mixture was denatured at 95°C for 5 min and analyzed using 12.5% denaturing PAGE. The PAGE gel was visualized by a fluorescent scanner (Figure S2A).

### 3.2 Preparation of modified dsDNA bearing one silicon rhodamine modification by primer extension (semi-preparative scale)

The reaction mixture (50 µl) containing primer (for sequence see Table S2; Prim^PEX^, 100 µM, 2.5 µl), template (for sequence see Table S2; Temp^PEX^, 100 µM, 2.5 µl), dGTP (4 mM, 1 µl), dC^SiR^TP (4 mM, 2.5 µl), KOD XL DNA polymerase (recombinant *Thermococcus kodakaraensis*, Cat.No: 71087, Merck Millipore, Germany) (2.5 U/µl, 1.5 µl) in corresponding reaction buffer (10×, 5 µl) supplied by the manufacturer. The reaction mixture was incubated for 75 min at 60°C in a thermal cycler. The reaction was stopped by cooling at 4°C. The modified dsDNA was purified using spin columns (QIAquick® Nucleotide Removal Kit, QIAGEN) and eluted by milli-Q water. Prepared dsDNA (Table S3) was used for the measurement of photophysical properties (Table S1, Figure S1). The product was analyzed using MALDI-TOF MS; calculated for [M]: 6942.2 Da; found: 6940.9 Da.

### 3.3. Enzymatic incorporation of dC^SiR^TP by polymerase chain reaction

The reaction mixture (20 µl) contained primer (for sequences see Table S2; Prim1^PCR^ and Prim2^PCR^, 10 µM, 1 µl of each), template (Temp^PCR^, 1 nM, 0.5 µl), natural dNTPs (dATP, dGTP, dTTP, 0.4 mM each, 1.5 µl) and either dCTP (0.4 mM, 1.5 µl), dC^SiR^TP (0.4 mM, 1.5 µl) or mixture of dC^SiR^TP with natural dCTP (0-95% of dCTP), KOD XL DNA polymerase (2.5 U/µl, 0.5 µl) and corresponding reaction buffer (10×, 2 µl) supplied by the manufacturer. After the initial denaturation for 3 min at 94 °C, 40 PCR cycles were run under the following conditions: denaturation for 20 sec. at 94°C, annealing for 30 sec. at 58 °C, extension for 30 sec. at 72 °C. The reaction was stopped by cooling to 4 °C. The PCR products were analyzed by agarose gel electrophoresis in 2% agarose gel stained with GelRed™ (Biotium, agarose gels are shown in Figure S2B).

## Table S2. Table of oligonucleotide sequences used in enzymatic synthesis.

| **Oligonucleotide** | **Length** | **Sequence (5´→3´)** |
| --- | --- | --- |
| Prim^PEX^ | 15-mer | 5´-CATGGGCGGCATGGG-3´ |
| Prim^PEX^-FAM ^(a)^ | 15-mer | 5´-FAM-CATGGGCGGCATGGG-3´ |
| Temp^PEX^ | 19-mer | 5´-CCCGCCCATGCCGCCCATG-3´ |
| Prim1^PCR^ | 20-mer | 5´-GACATCATGAGAGACATCGC-3´ |
| Prim2^PCR^ | 25-mer | 5´-CAAGGACAAAATACCTGTATTCCTT-3´ |
| Temp1^PCR^ | 98-mer | 5´-GACATCATGAGAGACATCGCCTCTGGGCTAATAGGACTA  CTTCTAATCTGTAAGAGCAGATCCCTGGACAGGCAAGGA  ATACAGGTATTTTGTCCTTG-3´ |

Underlined: segments of templates complementary to primers.
 ^a^ 6-carboxyfluorescein (6-FAM) used for oligonucleotide labeling at 5'-end.

## Table S3. Oligonucleotide prepared, isolated and characterized by MALDI-TOF mass spectrometry.

| **Oligonucleotide** | **Sequence (5´→3´). C*: modified nucleotide.** | **M_calculated_ (Da)** | **M_found_ (Da)** |
| --- | --- | --- | --- |
| **ON19_1C^SiR^** | 5´-CATGGGCGGCATGGGC*GGG-3´ | 6942.19 | 6940.9 |

##
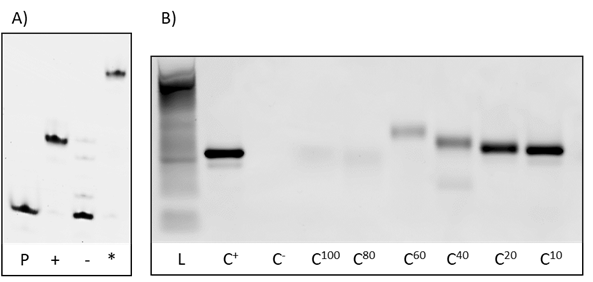


## Figure S2. (A) Page analysis of PEX using KOD XL polymerase, dC^SiR^TP and template Temp^PEX^. Primer (P), positive control (+, PEX with all natural dNTPs), negative control (-, PEX in absence of dCTP), PEX with dC^SiR^TP (*). (B) Agarose gel electrophoresis analysis of PCR amplification of 98 bp template Temp^PCR^ with KOD XL DNA polymerase using dC^SiR^TP. DNA ladder (L), primer (P), positive control (C^+^, PCR with natural dNTPs), negative control (C^-^, PCR in absence of dCTP), PCR with a mixture of modified nucleotide with natural dCTP (C^100^-C^10^, the content of modified nucleotide decreases from 100% to 10%).

## 4. Copy of ^1^H, ^13^C and ^31^P NMR spectra (dC^SiR^TP)

#
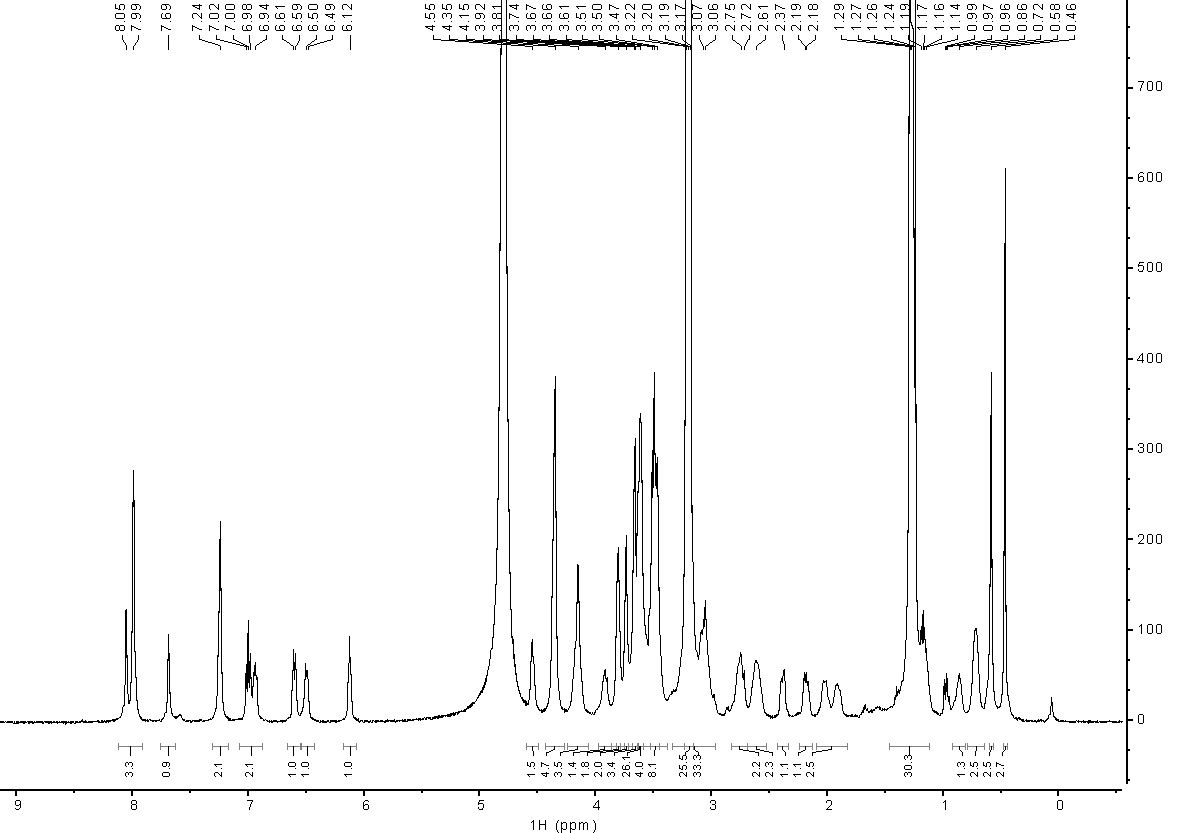


### Figure S3. ^1^H NMR spectrum of dC^SiR^TP in D_2_O.

###
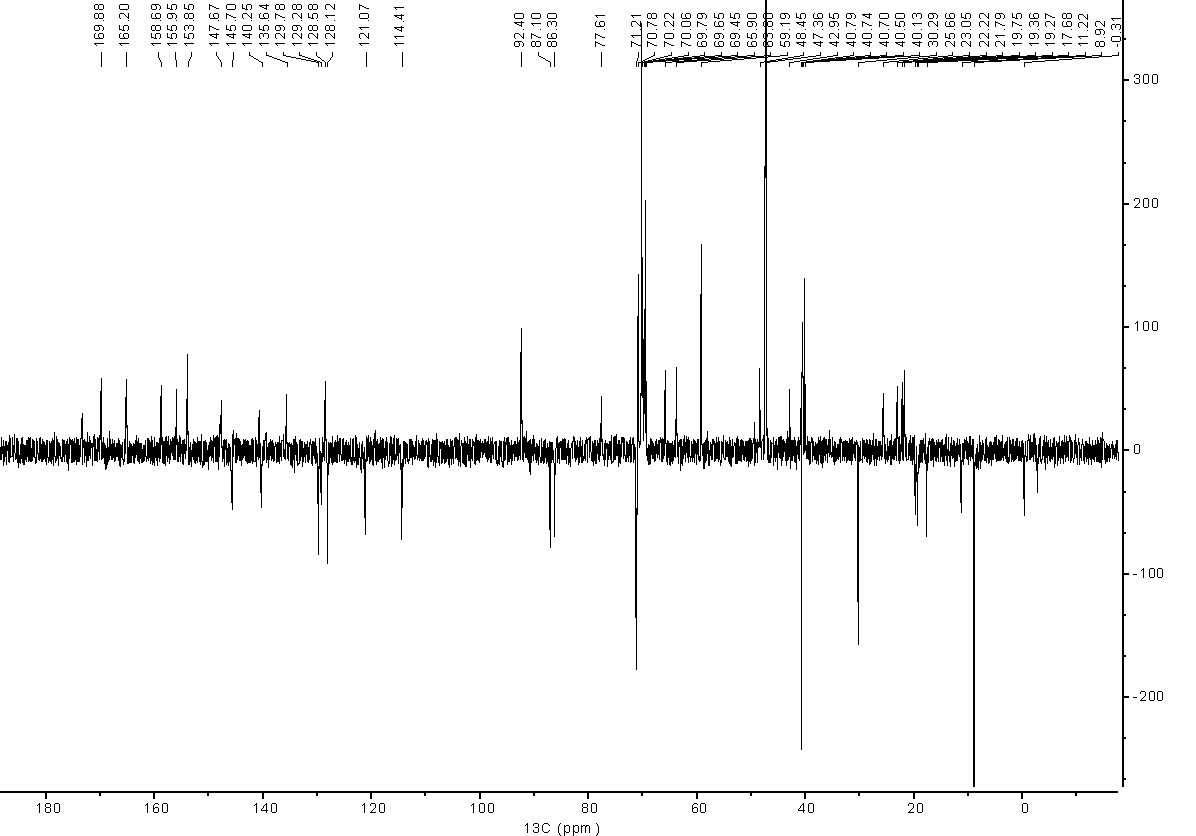


### Figure S4. ^13^C NMR spectrum of dC^SiR^TP in D_2_O.

###
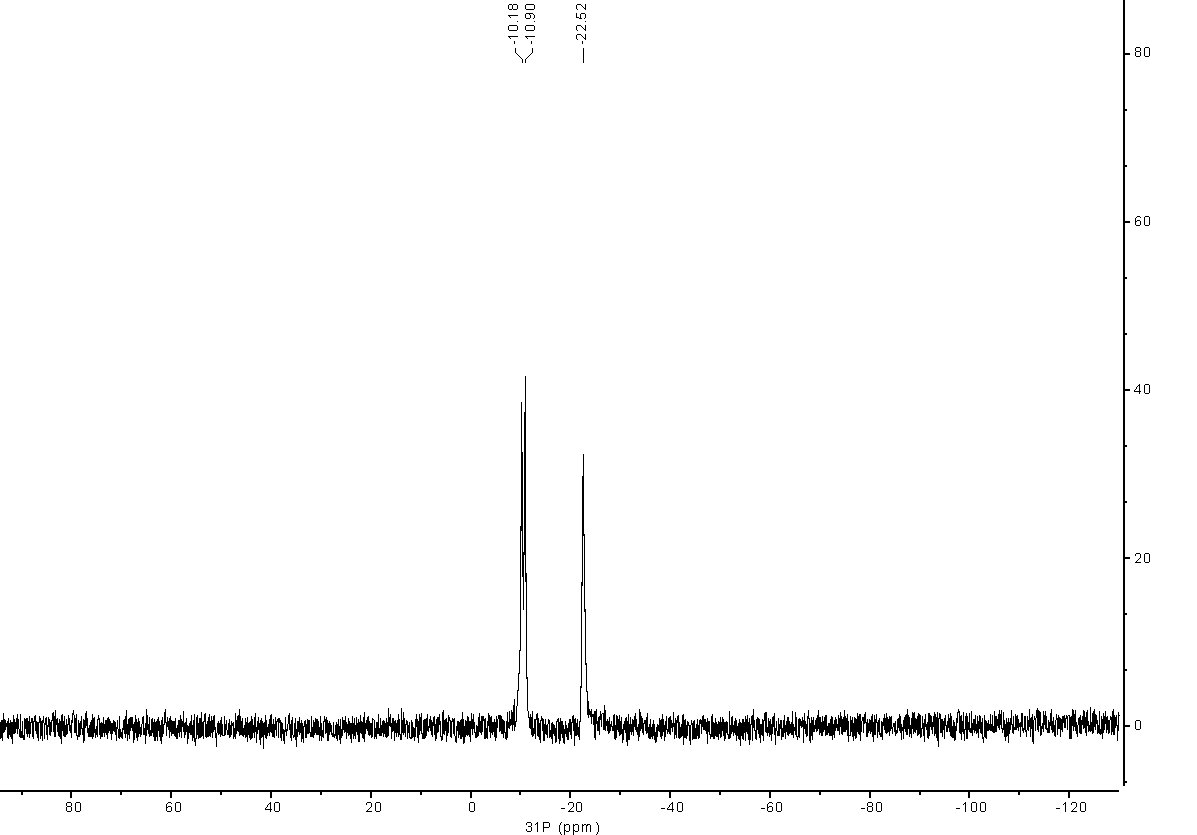


### Figure S5. ^31^P{^1^H}NMR spectrum of dC^SiR^TP in D_2_O.

# 5. Copy of mass spectra

#
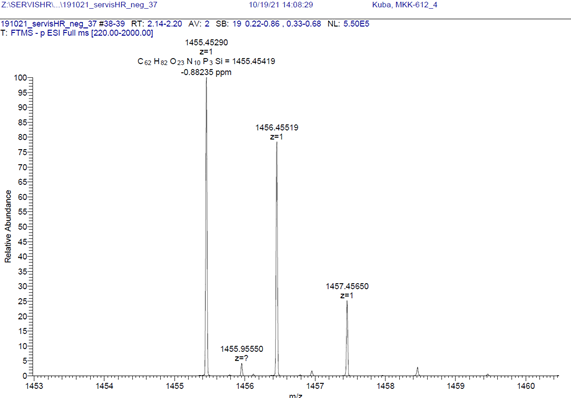


### Figure S6. ESI MS (neg. mode) high resolution spectra of dC^SiR^TP.

###
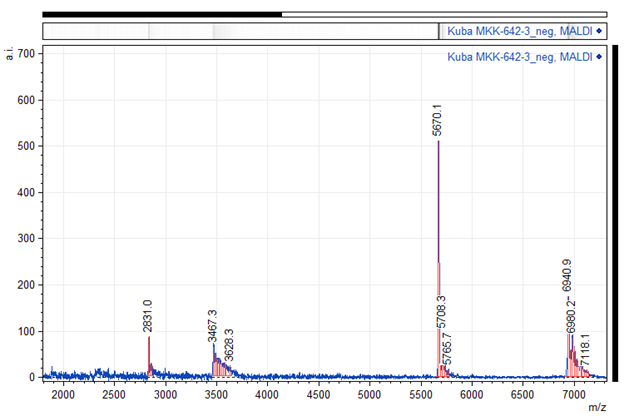


### Figure S7. MALDI-TOF mass spectrometry spectrum of single-modified PEX product (ON16_1CSiR) obtained using dCSiRTP. Calculated for [M]: 6942.2 Da; found: 6940.9 Da. The peak at m/z = 5670.1 Da represents template Temp1PEX.

### Table S4. Cell line characteristics

| **Name** | **Species** | **Type** | **Gender** | **Reference** |
| --- | --- | --- | --- | --- |
| hiPSC B4 | Homo sapiens | iPSC from human neonatal foreskin fibroblast (HFF1) | Male | [^[3]^](https://sciwheel.com/work/citation?ids=10884861&pre=&suf=&sa=0) |
| NSC B4 | Homo sapiens | Human neural stem cells | Male | This study |

### Table S5. Nucleotide and chemical characteristics

| **Name** | **Application** | **Detection** | **Cat #** | **Company** |
| --- | --- | --- | --- | --- |
| dC^SiR^TP | Replication labeling  (Labeling of nascent DNA) | - | - | This study |
| NTP-Transporter  Live Cell Dye (SNTT 1) | Transport of dC^SiR^TP across cell membrane of living cells | - | SCT064 | Merck, Germany |
| EdU  (5-ethynyl-2'-deoxyuridine) | Replication labeling  (Labeling of nascent DNA) | Click-IT | 7845.1 | Carl Roth, Germany |
| Abberior DNA live 590 dye | Live cell labeling of total DNA | - | LV 590-0143 | Abberior, Germany |
| Abberior Tubulin live 610 dye | Live cell labeling of tubulin | - | LV 610-0141 | Abberior, Germany |

### Table S6. Imaging systems characteristics

| **Microscope/**  **Company** | **Lasers/lamps** | **Filters (ex. &**  **em. [nm])*** | **Objectives/**  **lenses** | **Detection**  **system** | **Incubation**  **system** | **Application** |
| --- | --- | --- | --- | --- | --- | --- |
| Zeiss LSM 900 Airyscan, Zeiss, Germany | Four LEDs - (LED-Modul 385 nm, LED-Modul 470nm, LED-Modul 540-580 nm, LED-Modul 625 nm)  Laser - Power  405 nm- 5 mW  488 nm- 10 mW  561 nm- 10 mW  640 nm- 5 mW | ex: 390/40  em: 450/40 &  ex: 470/40  em: 525/50 &  ex: 550/25  em: 605/70  &  ex: 640/30  em: 690/50  Quad BP 425/30 + 514/30 + 592/25 + 709/100 | EC Plan-  Neofluar 2.5x  Plan-  Apochromat  20x  Plan-  Apochromat  40x  C Plan-  Apochromat  63x  alpha Plan-Apochromat 100x | Axiocam 820 mono SONY IMX541 CMOS back illuminated pixel size 2.74 μm x 2.74 μm 4512 x 4512 pixel QE: 86 % at 520 nm Full well capacity 10 000 e 20 fps full frame noise 2.3e at full well capacity GaAsP Detectors Additionally an Airyscan detector is available. | live cell incubation chamber for temperature, local humidity and CO2 are installed  Definite focus system for hardware focus stabilization  AI sample finder for automatic detection of the sample carrier, sample layout and sub-samples  photo manipulation is available through the regular user interface | Super resolution microscopy |
| Nikon TiE2 inverted with crest spinning disk unit/ Nikon, Japan | SPECTRA X light engine  395/25 nm with 295 mW  440/20 nm with 256 mW  470/24 nm with 196 mW  510/25 nm with 62 mW  540/30 nm with 231 mW  550/15 nm with 260 mW  575/25 nm with 310 mW | LED-DA/FI/TR/Cy5-4X-B  Quadbandpassex:390/18,  475/35,  535/50  em:460/60,  530/43,  580LP | 40x air (0.95 NA) & 250 µm WD*** | Cooled Nikon Qi2 camera and 16.25-megapixel sCMOS sensor. readout noise is: 2.2. electron | Self-build - 37°C incubation chamber, with 5% C02 and 60% humidity chamber | high throughput, high content imaging and image analysis |
| STED 775 QUAD Scan microscope (Abberior Instruments) | excitation laser pulsed diode laser 594 nm, <500mW (PDL 594, Abberior Instruments) pulsed diode laser 638nm, 20mW (PiL063X, Advanced Laser Diode Systems) | STED Laser pulsed fiber laser 775 nm, 1200mW (PFL-P-30- 775B1R, MPB Communications)  3D Module SLM based (easy 3D, Abberior Instruments) | 100 NA 1.4 Olympus UPlanSApo | Detectors:  APD: 605 nm - 625 nm  APD: 650nm - 720nm | - | Super resolution microscopy |
| Amersham AI600 imager | Chemiluminescen  ce, UV  transillumination | - | - | - | - | Western blots and  DNA agarose gels |

* ex.: excitation & em.: emission, ** dichroic specification, *** WD: working distance.

## 6. Generation of neural stem cells (NSC) and neural differentiation

hiPSC A4 and B4 were seeded at low density on p60 (Cat.No: 83.3901, Sarstedt, Germany) in hiPSC growth media (Methods: Cells) coated with vitronectin VTN 10 ng/ml (Cat.No.: A14700, Thermo Fisher Scientific, USA) for one hour. The cells were then allowed to grow for 6-7 days to form colonies. The cells were then treated with 1 mg/ml collagenase type IV (Cat.No: C4-28, Sigma Aldrich Chemie, Germany) in DMEM for 30 minutes until the colonies were detached and floating. The collagenase IV was deactivated using hiPSC growth media. The colonies with the media were then transferred to a 15 ml conical tube using a wide bore tip and were allowed to settle down for 10-15 minutes. The supernatant was aspirated slowly and resuspended in embryoid bodies formation media (Table S7) and were grown for 10 days in the media. The embryoid bodies were then transferred for attachment onto a 6 well plate coated with poly-L-ornithine / laminin in ITSF media and cultured for 10 days (Table S7). The attached embryoid bodies were disrupted using a glass hook and the cell clumps were transferred into a culture dish with no coating and resuspended into neurosphere media (Table S7). The neurospheres were cultured for another seven days. The neurospheres were then transferred into a 15 ml conical tube and were allowed to settle down for 10 - 15 minutes. The supernatant was then removed and 1 mL of accutase was added to the tube to dissociate the neurospheres. The cells were then resuspended in 5 mL neurosphere media, followed by centrifugation at 300 r.c.f for 5 minutes. The supernatant was removed, and cells were resuspended in 5 mL NSC media and transferred to poly-L-ornithine / laminin coated dishes. The NSCs were cultured and split every 5 days until the NSCs were obtained.

For differentiation experiments, NSCs were seeded on poly-L-ornithine / laminin coated dishes at 10^5^ cells per cm^2^ density. Next day, the media was replaced with neuronal differentiation media (Table S7). The cells were allowed to differentiate for 10 days before proceeding with live cell imaging.

**Table S7.** Media composition.

*Embryoid body media*

| **Name** | **Working solution** | **Cat #** | **Company** |
| --- | --- | --- | --- |
| IMDM (Iscove's Modified Dulbecco's Medium) | 75 % | 12440053 | Gibco, United States of America |
| Ham’s F12 nut mix | 25 % | 21765-029 | Gibco, United States of America |
| penicillin/streptomycin (100x) | 1 x | P0781 | Merck Millipore, Germany |
| N-2 supplement | 0.5 x | N2-K | Capricorn Scientific, Germany |
| B-27^TM^ supplement minus vitamin A | 0.5 x | 12587010 | Thermo Fisher Scientific, United States of America |
| knockout serum replacement | 0.05 % | 10828 | Gibco, United States of America |
| L-ascorbic acid | 50 µg/mL | A5960 | Sigma Aldrich, Germany |
| MTG (1-thioglycerol) | 4 x 10^-4^ M | M6145 | Sigma Aldrich, Germany |
| Recombinant Human BMP-IA/ALK-3 Fc Chimera | 500 ng/mL | 315-BR-100/CF | PeproTech, Germany |
| StemMACS™ SB431542 selective inhibitor of ALK5/TGF-𝛃 type I receptor) | 10 µM | SB431542 | Miltenyi Biotec, Germany |
| human-FGF2 | 10 ng/mL | 100-18G-250UG | PeproTech, Germany |

*ITSF media*

| **Name** | **Working solution** | **Cat #** | **Company** |
| --- | --- | --- | --- |
| DMEM / Ham's F12 nutrient mix | 100 % | 10565-018 | Gibco, United States of America |
| Apo-transferrin | 100 µg/mL | 616395 | Sigma Aldrich, Germany |
| Insulin | 25 µg/mL | I6634 | Sigma Aldrich, Germany |
| Fibronectin | 2.5 µg/mL | 33010018 | Thermo Fisher, United States of America |
| Sodium Selenite | 5 ng/mL | S5261 | Sigma Aldrich, Germany |
| penicillin/streptomycin (100x) | 1 x | P0781 | Merck Millipore, Germany |

*Neurosphere media*

| **Name** | **Working solution** | **Cat #** | **Company** |
| --- | --- | --- | --- |
| DMEM | 33.33 % | 41965039 | Gibco, United States of America |
| DMEM/ Ham's F12 Nut mix | 33.33 % | 10565-018 | Gibco, United States of America |
| Ham's F12 Nut mix | 33.33 % | 21765-029 | Gibco, United States of America |
| N-2 supplement | 0.5 x | N2-K | Capricorn Scientific, Germany |
| penicillin/streptomycin (100x) | 1 x | P0781 | Merck Millipore, Germany |
| h-FGF2 | 10 ng/mL | 100-18G-250UG | PeproTech, Germany |

*NSC media*

| **Name** | **Working solution** | **Cat #** | **Company** |
| --- | --- | --- | --- |
| DMEM | 33.33 % | 41965039 | Gibco, United States of America |
| DMEM/ Ham's F12 Nut mix | 33.33 % | 10565-018 | Gibco, United States of America |
| Ham's F12 Nut mix | 33.33 % | 21765-029 | Gibco, United States of America |
| N-2 supplement | 1 x | N2-K | Capricorn Scientific, Germany |
| B-27^TM^ supplement minus vitamin A | 0.5 x | 12587010 | Thermo Fisher Scientific, United States of America |
| Insulin | 20 µg/mL | I6634 | Sigma Aldrich, Germany |
| penicillin/streptomycin (100x) | 1 x | P0781 | Merck Millipore, Germany |
| human-EGF | 10 ng/mL | 100-15 | PeproTech, Germany |
| human-FGF2 | 10 ng/mL | 100-18G-250UG | PeproTech, Germany |

*Differentiation media*

| **Name** | **Working solution** | **Cat #** | **Company** |
| --- | --- | --- | --- |
| Neurobasal media | - | 21103049 | Thermo Fisher Scientific, United States of America |
| B-27^TM^ supplement minus vitamin A | 1 x | 12587010 | Thermo Fisher Scientific, United States of America |
| penicillin/streptomycin (100x) | 1 x | P0781 | Merck Millipore, Germany |
| Glutamax | 1 x | 35050038 | Thermo Fisher Scientific, United States of America |

### Table S8. Primary and secondary antibody characteristics

| **Reactivity** | **Host** | **Dilution** | **Cat #** | **Company** |
| --- | --- | --- | --- | --- |
| Sox2 | rabbit | 1:500 | #ab92494 /  EPR3131 | Abcam, United Kingdom |
| Oct3/4 | mouse | 1:500 | Clone 40/Oct-3 (RUO) | BD biosciences, United States of America |
| tubulin beta 3 | mouse | 1:200 | TUJ1 MAB1195 | Bio Techne GmbH, Germany |
| MAP2 | goat | 1:1000 | A104327 | antibodies.com, United Kingdom |
| GFAP | rabbit | 1:200 | ab7260 | Abcam, United Kingdom |
| Nestin | mouse | 1:200 | ab11300 | Abcam, United Kingdom |
| DCX | goat | 1:100 | ab113435 | Abcam, United Kingdom |
| Pax6 | rabbit | 1:1000 | ab5790 | Abcam, United Kingdom |
| NeuN | rabbit | 1:500 | EPR12763 | Abcam, United Kingdom |
| lamin B1 | mouse | 1:10 | X 223 (65147C) | Progen Biotechnik GmbH, Germany |
| Ki67 | rabbit | 1:500 | ab16667 | Abcam, United Kingdom |
| Histone 3 | rat | 1:1000 | 1C8B2 (61647) | Active Motif, Carlsbad, CA, USA |
| H3 K9 me3 | rabbit | 1:500 | 39161 | Active Motif, Carlsbad, CA, USA |
| H3 K36 me3 | rabbit | 1:500 | ab9050 | Abcam, United Kingdom |
| H3 K27 me3 | mouse | 1:500 | MABI 0323 | Active Motif, Carlsbad, CA, USA |
| H3 K9 ac | rabbit | 1:500 | 39917 | Active Motif, Carlsbad, CA, USA |
| ɑ-mouse IgG A488-conjugated | goat | 1:500 | A11029 | Thermo Fisher Scientific, USA |
| ɑ-mouse IgG Cy3-conjugated | donkey | 1:500 | JIM-715-165-150 | Jackson ImmunoResearch Europe Ltd., United Kingdom |
| ɑ-mouse IgG Cy5-conjugated | donkey | 1:500 | JIM-715-175-150 | Jackson ImmunoResearch Europe Ltd., United Kingdom |
| ɑ-rabbit IgG A488-conjugated | donkey | 1:500 | A11034 | Thermo Fisher Scientific, USA |
| ɑ-rabbit IgG Cy3-conjugated | donkey | 1:500 | JIM-711-165-152 | Jackson ImmunoResearch Europe Ltd., United Kingdom |
| ɑ-rabbit IgG Cy5-conjugated | donkey | 1:500 | JIM-711-175-152 | Jackson ImmunoResearch Europe Ltd., United Kingdom |
| ɑ-rat IgG Cy5-conjugated | donkey | 1:500 | A10525 | Thermo Fisher Scientific, USA |
| ɑ-goat IgG A568-conjugated | donkey | 1:500 | A-11057 | Thermo Fisher Scientific, USA |

**Table S9.** Software

| **Name** | **Version** | **Website** | **Company/University** | **Application** |
| --- | --- | --- | --- | --- |
| Volocity | 6.3 | - | PerkinElmer, USA | Analysis of DNA domain sizes |
| Fiji | 1.53c | <https://imagej.net/software/fiji/> | Wayne Rasband, National Institutes of Health, USA | Image processing and image analysis |
| RStudio | 1.1.447-1.2.5033 | <https://rstudio.com/> | RStudio | Statistical analysis and plotting |
| KNIME Analytics | 3.5.2 | https://www.knime.com/knime-analytics-platform | KNIME AG, Switzerland | High content microscopy image processing and analysis |
| Adobe Illustrator | 2025 | <https://www.adobe.com/> | Adobe, USA | Graphical sketch and figures arrangement |
| Zeiss Zen | 3.9 | https://www.zeiss.com/microscopy/de/produkte/software/zeiss-zen.html | Zeiss, Germany | Super resolution processing of time lapse microscopy raw data |


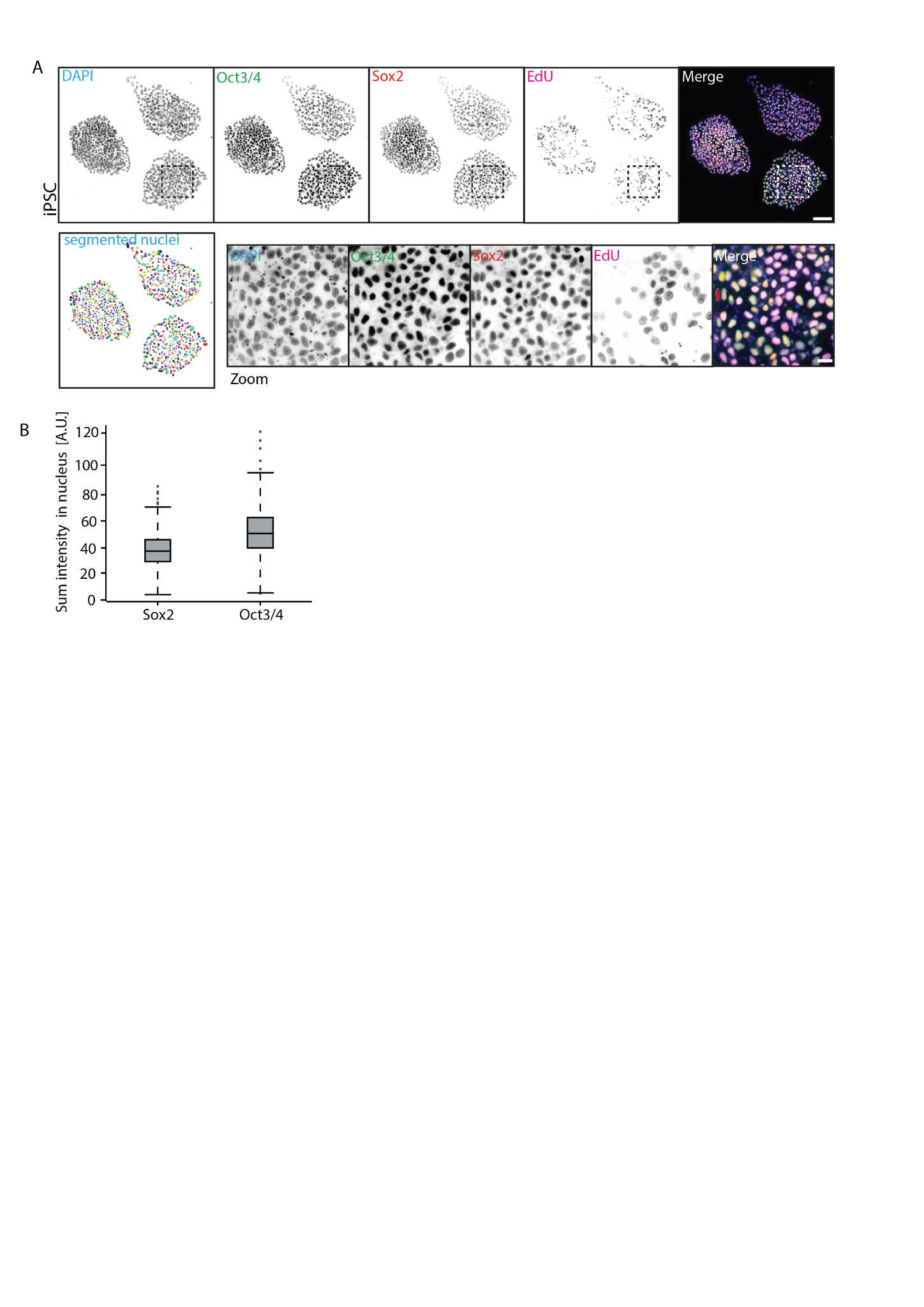
**Figure S8.** **(A)** Immunofluorescence detection and quantification of pluripotent markers (Oct3/4 and Sox2) and detection of replicating cells using EdU (10 minutes, 10 µm) nucleoside pulse in iPSC cells (Methods). **(B)** Quantification of sum nuclear intensity of Sox2 and Oct3/4 quantified and represented as box plots. Scale bars: 50 µm.

###
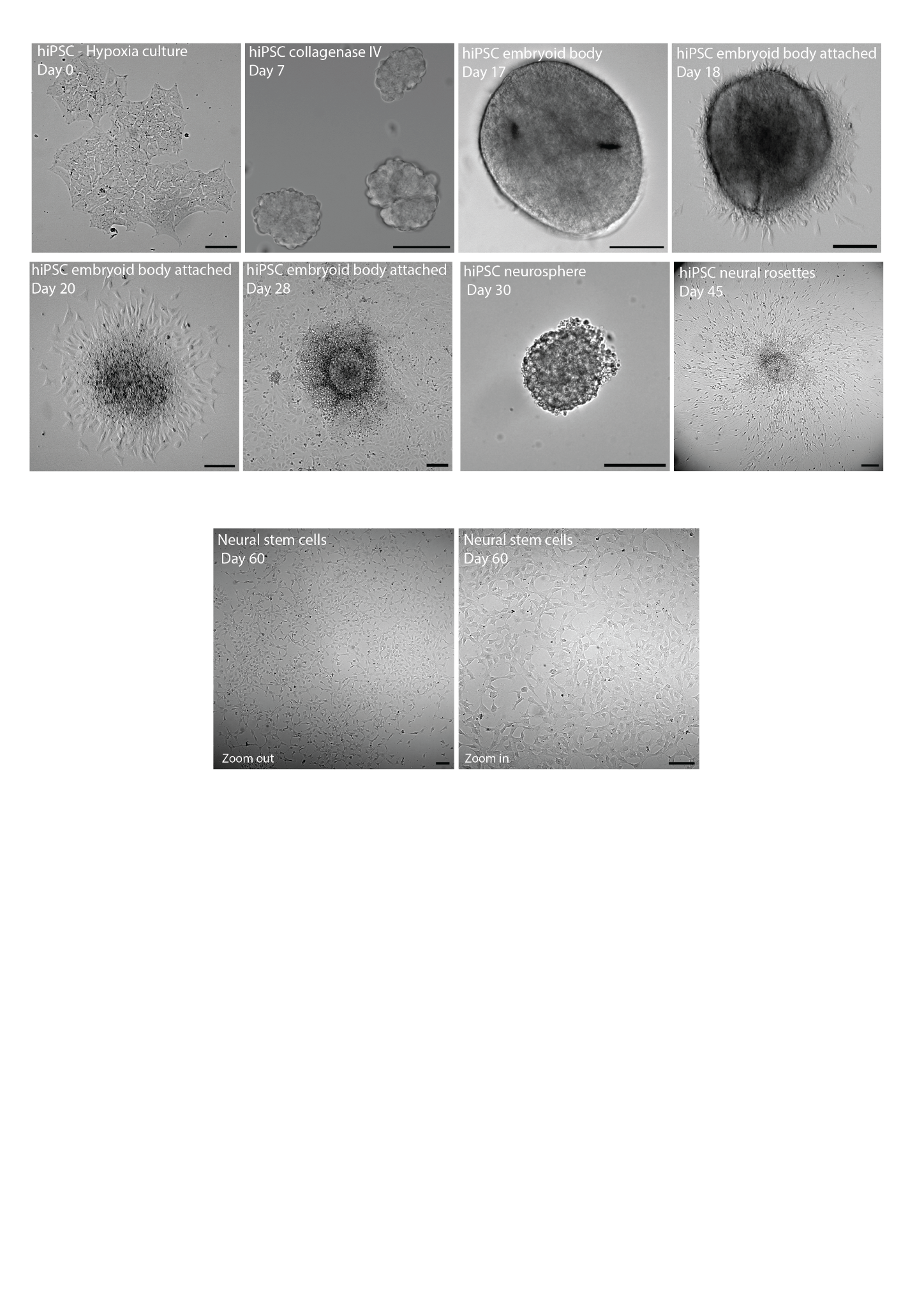


**Figure S9.** Representative images using differential interference contrast (DIC) microscopy of live cells during the generation of neural stem cells (NSCs) from induced pluripotent stem cells (iPSCs). Scale bars: 100 µm.

###
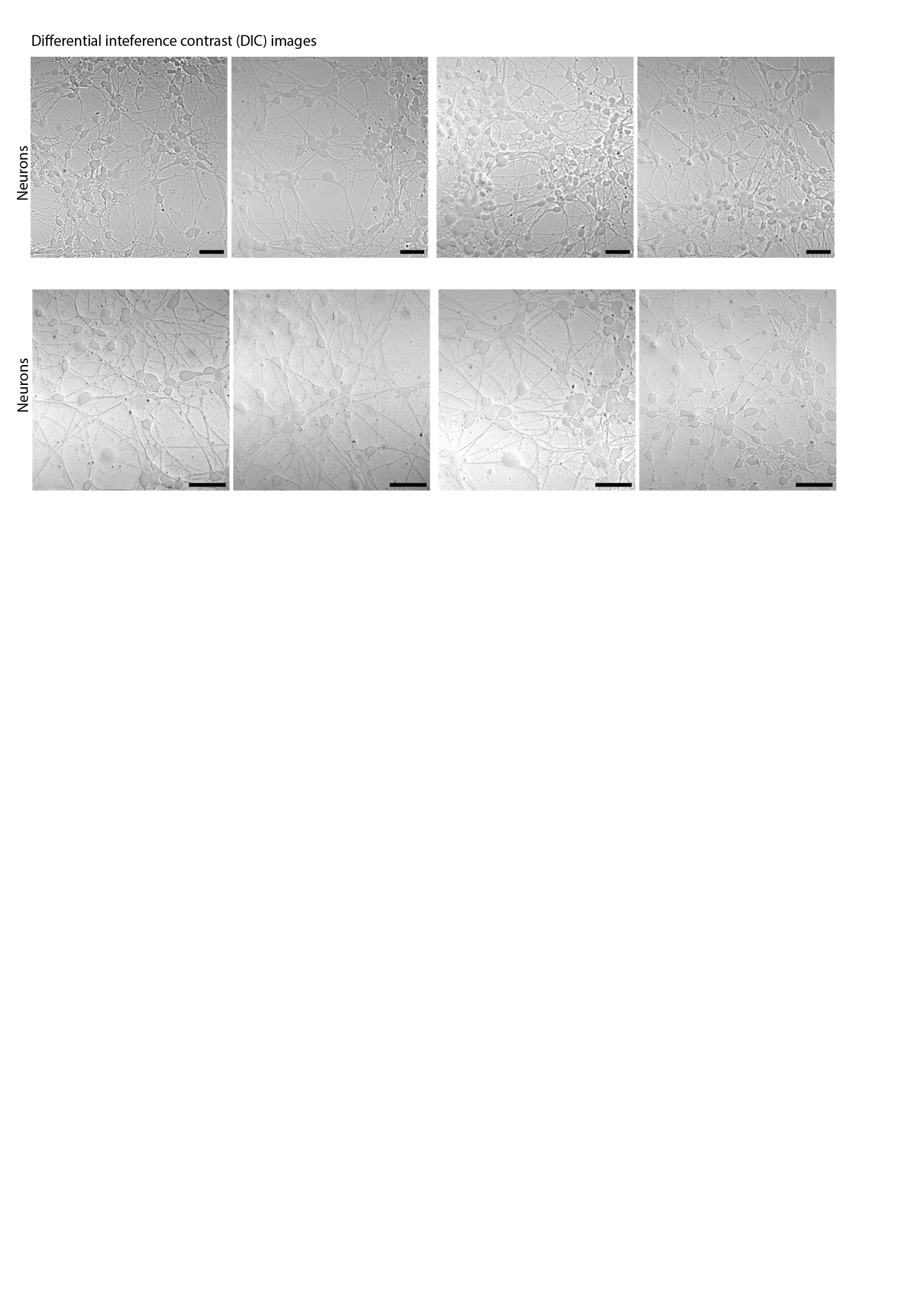


**Figure S10.** Differential interference contrast (DIC) representative images of differentiated neurons. Scale bars: 25 µm.


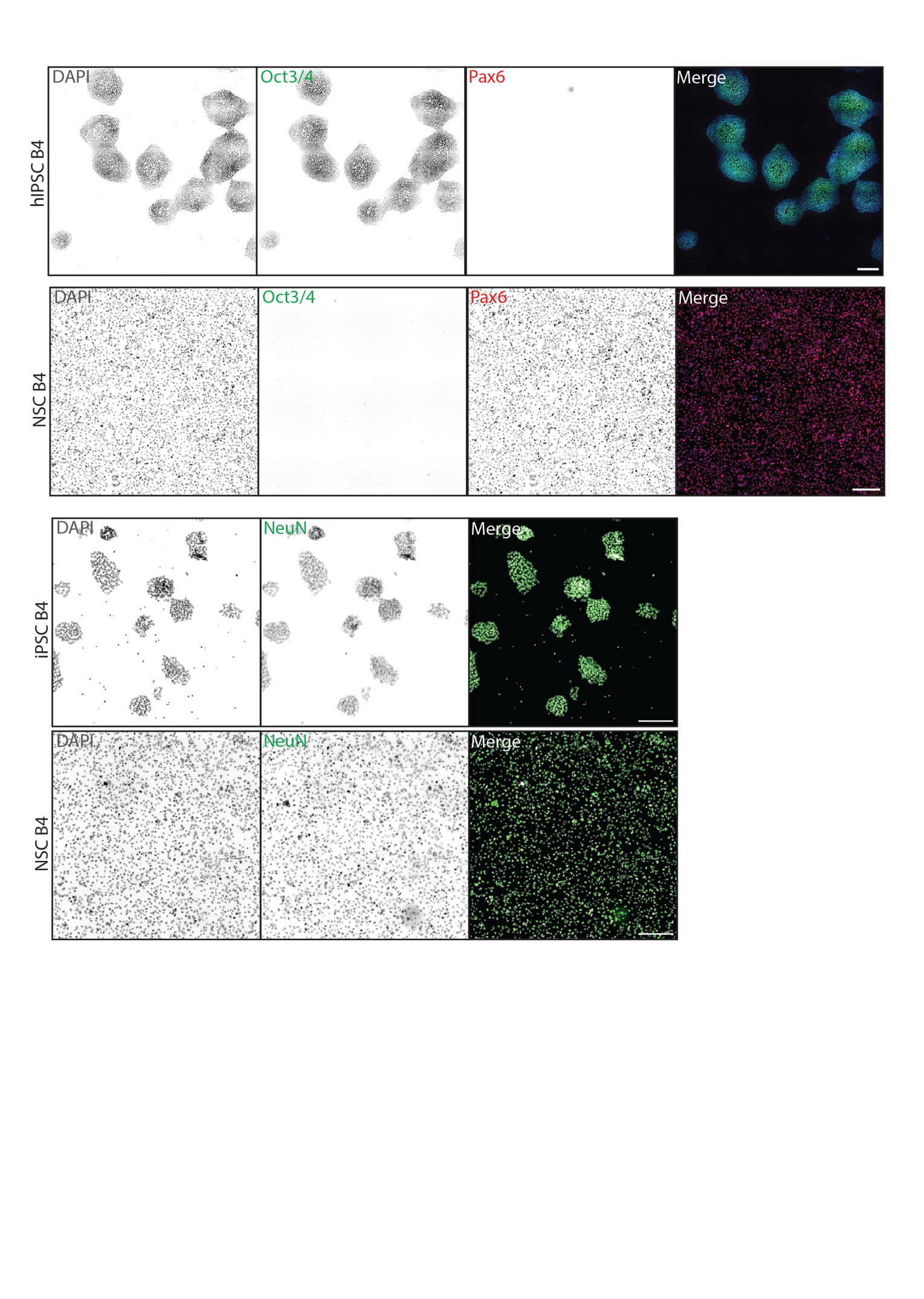
**Figure S11.** Immunofluorescence detection of pluripotent and neural stem cell markers on iPSCs and NSCs (Table S8, Methods: Immunofluorescence). Scale bars: 250 µm.

###

###

**
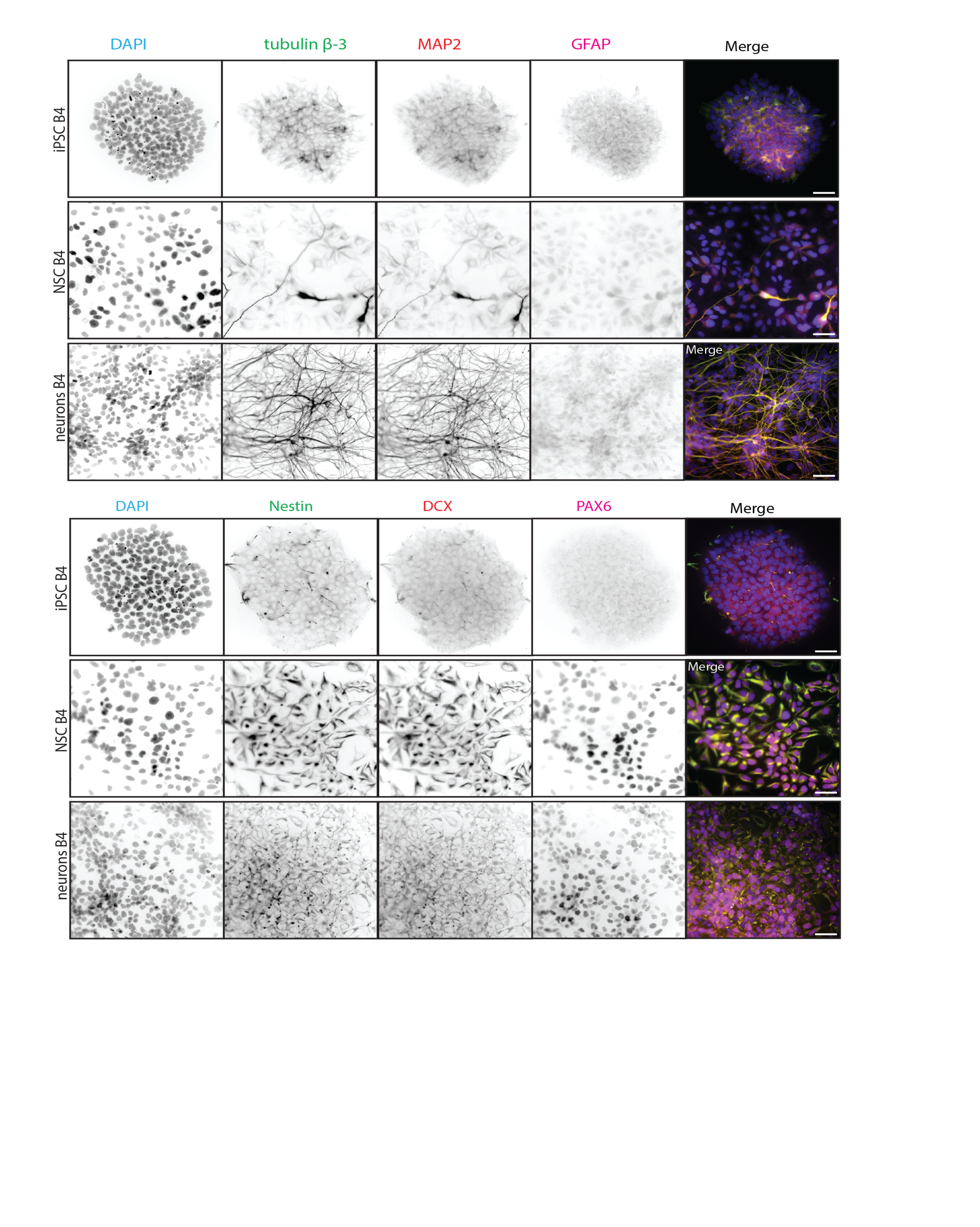
**

**Figure S12.** Immunofluorescence detection of neuronal markers on iPSCs, NSCs, and neurons (Table S8, Methods). Scale bars: 50 µm.


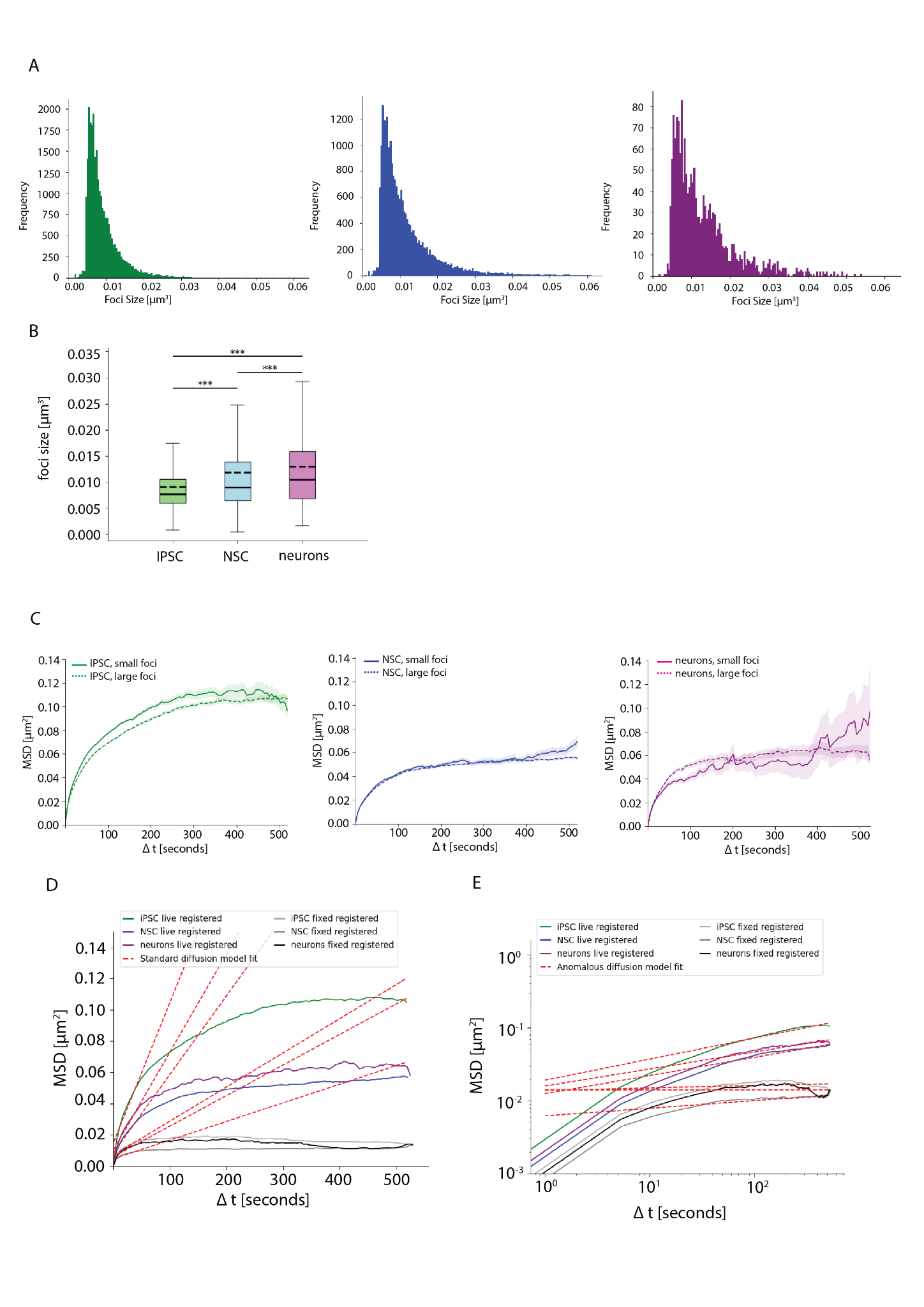


**Figure S13.** **(A)**The size distribution of labelled chromatin domains for IPSC (green), NSC (blue), and neurons (magenta) were plotted as a histogram with frequency as y-axis and volume of foci (µm^3^). **(B)** The boxplot for volume of labelled chromatin domains foci (µm^3^) was plotted with median (solid line) and mean (dash line) and significance values (*** is p ≤ 0.001). **(C)** Mean square displacement (y-axis, µm^2^) over time (x-axis, Δt in seconds) for iPSC (green), NSC (blue), and neurons (magenta) for small and large foci. The median foci size of a trajectory was determined and the foci were distinguished into small and large by having an even split (same number of trajectories). **(D)** Mean square displacement curves for iPSC (green), NSC (blue), neurons (magenta), and fixed cells (gray shades). The standard diffusion model fitted to the linear part at the beginning of the MSD curves (short-range diffusion) is plotted in red. **(E)** Mean square displacement curves for iPSC (green), NSC (blue), neurons (magenta), and fixed cells (gray shades) on a log-log scale. The anomalous diffusion model fitted to the whole MSD curves is plotted in red.

###

### 7. Supplementary movies

**Movie 1**. Live cell time lapse imaging of chromatin labeling in iPSC. Scale bar: 50 µm.

**Movie 2.** Live cell time lapse imaging of labeled chromatin in iPSC, NSC, neurons. Scale bar: 5 µm.

**Movie 3.** Registered and non-registered timelapses of labeled chromatin in iPSC, NSC, neurons. Scale bar: 5 µm.

**Movie 4.** Live and fixed cell time lapse imaging in iPSC. Scale bar: 50 µm.

**References**

[[1]    M. Kuba, P. Khoroshyy, M. Lepšík, E. Kužmová, D. Kodr, T. Kraus, M. Hocek; Real‐time Imaging of Nascent DNA in Live Cells by Monitoring the Fluorescence Lifetime of Dna‐incorporated Thiazole Orange‐modified Nucleotides. *Angew. Chem.* **2023**, August. doi:10.1002/ange.202307548.](https://sciwheel.com/work/bibliography/16862167)

[[2]    C. Würth, M. Grabolle, J. Pauli, M. Spieles, U. Resch-Genger; Relative and Absolute Determination of Fluorescence Quantum Yields of Transparent Samples. *Nat. Protoc.* **2013**, 8, 1535–50. doi:10.1038/nprot.2013.087.](https://sciwheel.com/work/bibliography/5424300)

[[3]    Y. Wang, J. Adjaye; A Cyclic AMP Analog, 8-Br-CAMP, Enhances the Induction of Pluripotency in Human Fibroblast Cells. *Stem Cell Rev.* **2011**, 7, 331–41. doi:10.1007/s12015-010-9209-3.](https://sciwheel.com/work/bibliography/10884861)
